# Rapid Detection of SARS-CoV-2 Variants Using an Angiotensin-Converting Enzyme 2-Based Surface-Enhanced Raman Spectroscopy Sensor Enhanced by CoVari Deep Learning Algorithms

**DOI:** 10.1021/acssensors.4c00488

**Published:** 2024-06-06

**Authors:** Yanjun Yang, Jiaheng Cui, Dan Luo, Jackelyn Murray, Xianyan Chen, Sebastian Hülck, Ralph A. Tripp, Yiping Zhao

**Affiliations:** † School of Electrical and Computer Engineering, College of Engineering, The University of Georgia, Athens, Georgia 30602, United States; ‡ Department of Statistics, The University of Georgia, Athens, Georgia 30602, United States; § Department of Infectious Diseases, College of Veterinary Medicine, The University of Georgia, Athens, Georgia 30602, United States; ∥ Department of Epidemiology & Biostatistics, College of Public Health, The University of Georgia, Athens, Georgia 30602, United States; ⊥ Tec5USA Inc., Plainview, New York 11803, United States; # Department of Physics and Astronomy, The University of Georgia, Athens, Georgia 30602, United States

**Keywords:** surface-enhanced Raman scattering (SERS), silver nanorod array, SARS-CoV-2 detection, angiotensin-converting enzyme 2 (ACE2), deep learning, convolutional neural network

## Abstract

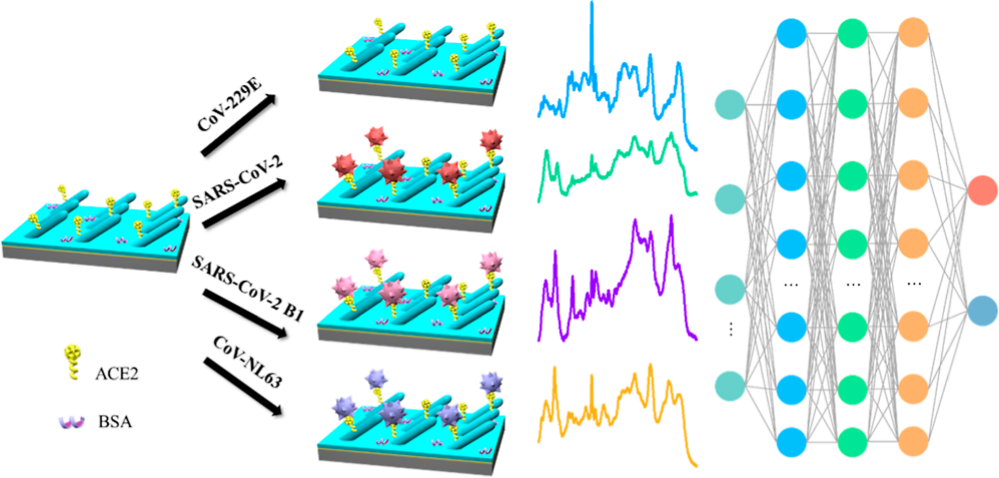

An integrated approach combining surface-enhanced Raman spectroscopy (SERS) with a specialized deep learning algorithm to rapidly and accurately detect and quantify SARS-CoV-2 variants is developed based on an angiotensin-converting enzyme 2 (ACE2)-functionalized AgNR@SiO_2_ array SERS sensor. SERS spectra with concentrations of different variants were collected using a portable Raman system. After appropriate spectral preprocessing, a deep learning algorithm, CoVari, is developed to predict both the viral variant species and concentrations. Using a 10-fold cross-validation strategy, the model achieves an average accuracy of 99.9% in discriminating between different virus variants and *R*^2^ values larger than 0.98 for quantifying viral concentrations of the three viruses, demonstrating the high quality of the detection. The limit of detection of the ACE2 SERS sensor is determined to be 10.472, 11.882, and 21.591 PFU/mL for SARS-CoV-2, SARS-CoV-2 B1, and CoV-NL63, respectively. The feature importance of virus classification and concentration regression in the CoVari algorithm are calculated based on a permutation algorithm, which showed a clear correlation to the biochemical origins of the spectra or spectral changes. In an unknown specimen test, classification accuracy can achieve >90% for concentrations larger than 781 PFU/mL, and the predicted concentrations consistently align with actual values, highlighting the robustness of the proposed algorithm. Based on the CoVari architecture and the output vector, this algorithm can be generalized to predict both viral variant species and concentrations simultaneously for a broader range of viruses. These results demonstrate that the SERS + CoVari strategy has the potential for rapid and quantitative detection of virus variants and potentially point-of-care diagnostic platforms.

Surface-enhanced Raman spectroscopy (SERS) has recently gained significant attention as a promising diagnostic platform for detecting SARS-CoV-2.^1^ This interest stems from its exceptional sensitivity, ability to generate unique spectral features specific to different viruses, inherent simplicity, and the potential for developing point-of-care detection devices.^2,3^ Various detection strategies have emerged, such as direct detection of viral particles,^4−6^ RNA hybridization,^7,8^ spike protein capture and detection,^9^ and SERS tag labeling.^10,11^ While label-free detection strategies are straightforward, the presence of a body fluid matrix, buffer, or other processing fluids introduces complications due to interference from contaminants and other biomolecules, making spectral analysis challenging. Although deep learning provides a powerful tool for spectrum analysis, large variations in spectra require extensive training data, covering all possible spectra to learn the patterns and relationships, which can be challenging due to factors such as variations in spectral signal-to-noise ratio, diversity in the instrumentation and measurement conditions, and the complexity of the specimens. To enhance specificity, capture-based methods have been developed using functionally designed agents to target virus-specific biomolecules. For instance, DNA probes have been designed to hybridize specifically with SARS-CoV-2 RNA.^8^ However, subtle changes in SERS signals after RNA hybridization pose challenges, especially for differentiating virus variants. To address this, multiple DNA probes for distinct SARS-CoV-2 variants need to be developed, or a reliable spectral analysis method to differentiate minute changes after RNA hybridization must be established. Nevertheless, functionalizing a SERS sensor to capture a target analyte from complicated specimens accurately can enhance the SERS response and ensure a reproducible diagnosis.

Detecting SARS-CoV-2 variants is crucial for understanding transmission, assessing vaccine efficacy, and adapting public health measures. Since the beginning of the COVID-19 pandemic, SARS-CoV-2 has been circulating for a long time, with the accumulation of gene mutations and significant changes in the viral gene sequence leading to corresponding changes in acupuncture (S) proteins, viral transmission, and antigenicity.^12^ The most accurate SARS-CoV-2 variant detection method is polymerase chain reaction. To do so, one needs to understand the entire gene sequence of SARS-CoV-2 and design specific primers that can be used to replicate RNA and fluorescence tags to show detection signals, making it material-dependent and complex.^13^ Thus, a simpler, less material-dependent diagnostic method is desirable.

Angiotensin-converting enzyme 2 (ACE2) is the receptor through which the spike (S) protein of the SARS-CoV-2 virus binds to and facilitates its entrance to host cells.^14^ Studies have shown that variants in SARS-CoV-2 can replace the RBD amino acid sequence of the S-protein and affect the affinity between the S-protein and ACE2. Therefore, such a change in ACE2 binding affinity due to mutations in the RBD amino acid sequence of S-protein makes ACE2-based capture detection advantageous for identifying SARS-CoV-2 variants.^15^ Though there are several reports on ACE2-based SERS sensors,^9,16−19^ none of them take advantage of this. For example, Yang et al. used ACE2-modified Au nanospike arrays and demonstrated a low limit of detection (LOD) of 80 copies/mL for SARS-CoV-2 in contaminated wastewater within 5 min.^9^ They also demonstrated the selective capture and rapid detection of coronavirus-expressing S-protein. Payne et al. used ACE2 as a viral protein capture probe and a multivariable calibration model to identify and quantify the unique vibration characteristics of the surface modified by S-protein binding peptide and showed a detection limit of 300 nM.^16^ Zhang et al. used ACE2-modified AgNR substrates to test the availability of COVID-19 from 23 water samples against bacteria.^17^ Li et al.^18^ and Pramanik et al.^19^ used Fe_3_O_4_-based SERS substrates to collect and concentrate SARS-CoV-2 for SERS detection. Awada et al.^20^ and Yeh et al.^21^ recently focused on S-protein detection and achieved LODs of 1 fM and 1 fg/mL, respectively. However, none of these demonstrates variant detection or viral concentration quantification.

Here, we present an ACE2-functionalized AgNR@SiO_2_ array SERS sensor designed for detecting SARS-CoV-2 and its variants, including SARS-CoV-2, SARS-CoV-2 B1, and CoV-NL63. A CoVari deep learning algorithm is developed to predict both the variants and corresponding concentrations. A 10-fold cross-validation strategy is applied to demonstrate the robustness and reproducibility of the model. The cross-validation has achieved 99.9% average accuracy in discriminating between different variants and attained *R*^2^ values larger than 0.98 for quantifying variant concentrations. The permutation algorithm-based calculation of feature importance for both classification and regression gives a better understanding of the CoVari algorithm. These results demonstrate the high potential of the SERS + CoVari strategy for rapidly detecting SARS-CoV-2 variants, positioning it as a promising candidate for point-of-care diagnostic platforms.

## Experimental Section

**Figure 1:**
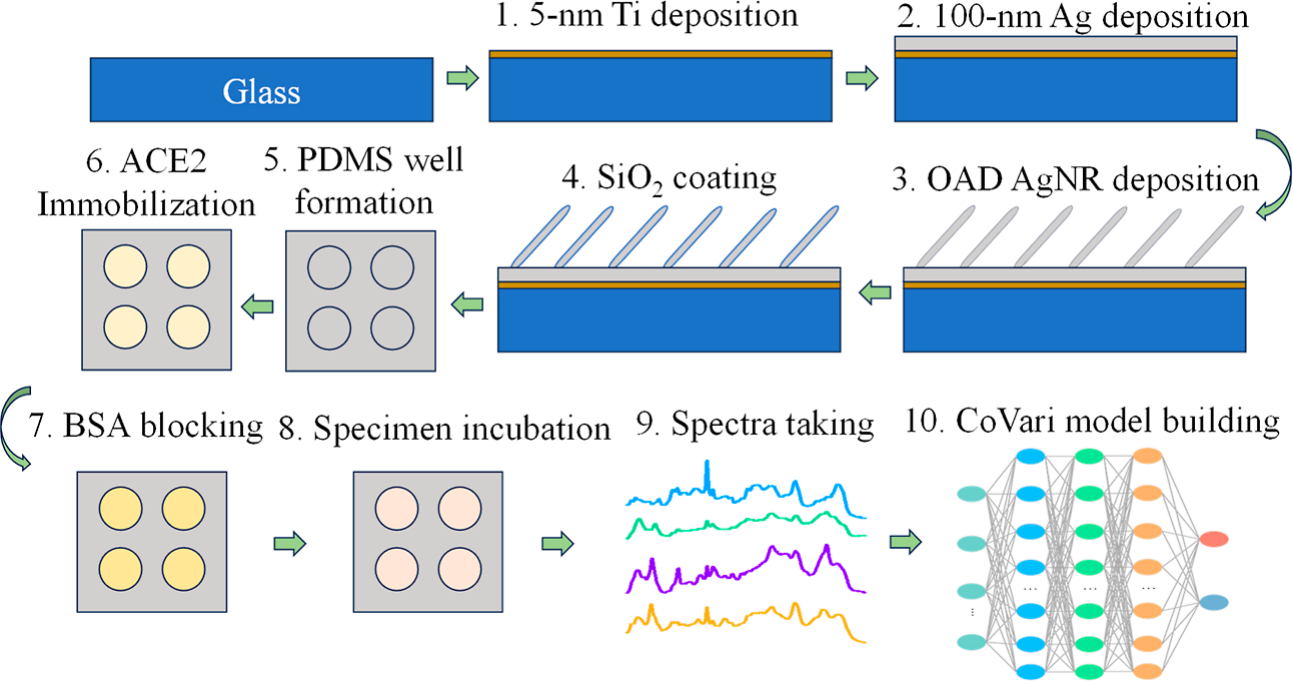
Schematic illustration of substrate fabrication and detection process for ACE2-based virus detection using SERS and the CoVari deep learning algorithm.

The general experimental procedures are shown in Figure 1, and they can be divided into 4 major steps: SERS substrate fabrication (steps 1–5), ACE2 functionalization (steps 6–7), spectra collection (steps 8–9), and the establishment of the CoVari deep learning algorithm (step 10).

### SERS Substrate Fabrication

AgNR arrays were prepared by the oblique angle deposition method, as previously described.^22−100^ The corresponding diagram of the setup is shown in Figure S1A of the Supporting Information. Briefly, clean glass slides (0.5 in. × 0.5 in.) were loaded into a custom-built vacuum deposition chamber with the substrate normal antiparallel to the incident vapor direction (θ = 0°). Substrate normal refers to a vector or direction perpendicular to the surface of glass substrates. A layer of 5 nm titanium (Ti, Kurt J. Lesker, 99.995%) and a subsequent layer of 100 nm silver (Ag, Kurt J. Lesker, 99.99%) films were deposited at a rate of 0.2 and 0.3 nm/s, respectively (steps 1–2 in Figure 1). Then, the substrate normal was rotated to an angle θ = 86° relative to the incident vapor direction, and a thickness of 2000 nm Ag film was then deposited at a rate of 0.3 nm/s to obtain the arrayed AgNRs (step 3). The evaporation process was conducted under a high vacuum (<3 × 10^–6^ Torr).

To mitigate the high reactivity of AgNR with buffers or biological fluids and improve the stability of AgNR substrates, an ultrathin oxide layer was coated on the nanorod surfaces using a simple hydrolysis method (step 4).^26^ In brief, AgNR arrays were immersed into a homogeneous mixture composed of 30 mL of ethanol (EtOH; Sigma-Aldrich, 95%), 4 mL of H_2_O, and 500 μL of tetraethyl orthosilicate (TEOS; Alfa Aesar, 99.9%) for 20 min under stirring. Once 560 μL of ammonium hydroxide (J. T. Baker, 28.0–30.0 wt %) was added to the mixture, the oxide coating on AgNR was initiated. The AgNR arrays were removed from the reaction solution after a 5 min immersion, followed by DI water rinsing and N_2_ drying. The estimated SiO_2_ coating thickness was 2 nm.^26^Figure S1B shows a representative SEM image of the AgNR@SiO_2_ array.

The AgNR@SiO_2_ substrates were patterned by a polymer-molding technique to provide a uniform array for high-throughput biosensing and multiplexing (step 5). A polydimethylsiloxane layer with arrayed small wells (2 × 2 wells, with a well diameter of 4 mm and a well depth of 1 mm) was molded on the AgNR@SiO_2_ array to restrict the effective sensing areas, referred to as AgNR@SiO_2_ wells.

### SERS Sensor Fabrication and Characterization

To fabricate ACE2-immobilized AgNR@SiO_2_ SERS substrates (AgNR@SiO_2_-ACE2) (step 6), 200 μL of 0.62 mg/mL ACE2 was dialyzed in phosphate buffer (100 mM Na_2_HPO_4_ and 100 mM NaH_2_PO_4_, pH = 7.4) and diluted to 124 μg/mL with phosphate buffer. Then, 20 μL of ACE2 dilute solution was transferred to each AgNR@SiO_2_ well and incubated for 2 h at room temperature. After incubation, the wells were washed with DI water 3 times. Subsequently, 20 μL of 1 mg/mL BSA solution was dispensed in each AgNR@SiO_2_-ACE2 well (step 7) and incubated for 2 h to block the ACE2-uncovered area of AgNR@SiO_2_ and avoid nonspecific binding of viral particles. Finally, the wells (we called them ACE2-well) were rinsed with DI water and air-dried.

### Virus Preparation

The following viruses were used in this study: SARS-CoV-2 (WA1/2020), SARS-CoV-2 B1.1.7 variant (SARS-CoV-2 B1), human coronavirus NL63 (CoV-NL63), and human coronavirus 229E (CoV-229E). All viruses were propagated in Vero E6 cells, which were maintained in Dulbecco’s modified Eagle’s medium (DMEM; GIBCO BRL laboratories, Grand Island, NY) supplemented with 1% fetal bovine serum (FBS; Hyclone Laboratories, Salt Lake City, UT). Cells were infected using a multiplicity of infection = 0.1. After 48 h, the viruses were harvested in serum-free DMEM followed by freeze–thaw, after which the contents were collected and centrifuged at 4000 g for 15 min at 4 °C. The virus titers were similar, i.e., 10^5^ PFU/mL, as determined by plaque assay as previously described.^27−29^ The reference buffer for these studies was DMEM supplemented with 1% FBS. All the experiments on SARS-CoV-2 and SARS-CoV-2 variants were conducted in a biosafety level 3 (BSL-3) lab, while others were performed in a BSL-2 lab. All the experimental operations followed the biosafety guidelines: https://www.cdc.gov/coronavirus/2019-nCoV/lab/lab-biosafety-guidelines.html.

### Concentration-Dependent Detection of Coronaviruses

Cell-free supernatant suspensions of CoV NL63, SARS-CoV-2, and SARS-CoV-2 B1 ranging from 98 to 10^5^ PFU/mL in PBS buffer were transferred into the ACE2-wells and incubated for 20 min at room temperature (step 8). Subsequently, the ACE2-wells were washed 3× with DI water and air-dried for SERS measurements.

### Concentration-Dependent Detection of Coronavirus Spike Proteins in Saliva

Three coronavirus spike protein variants (Sino Biological), including SARS-CoV-2 spike, SARS-CoV-2 spike (BA 2.75.2), and SARS-CoV-1 spike, ranging from 25.6 pg/mL to 50 μg/mL in saliva, were added into the ACE2-wells and incubated for 20 min at room temperature (step 8). Subsequently, the ACE2-wells were washed 3× with DI water and air-dried for SERS measurements.

### SERS Spectra Measurements

The SERS spectra were collected using the Tec5USA Raman spectrometer (step 9). The laser power was 32 mW, and the acquisition time was 1 s. The maximum SERS signal was obtained for each measurement by adjusting the *Z* stage. To collect a large amount of SERS spectra for deep learning algorithm training, the *XY* stages were tuned with a step of 0.3 mm to move the laser to different locations during the SERS measurements. More than 200 SERS spectra were collected from multiple randomly selected locations for every variant with every concentration. The numbers of SERS spectra collected from both references and viruses are summarized in Table S1, and the total spectra number is 12,545.

### Spectra Pretreatment

All the SERS spectra were preprocessed following a procedure that included despiking, baseline removal, and area normalization. We implemented the “Gaussian–Lorentzian function fitting” baseline removal method^4,30^ based on the overall spectral features of SERS spectra obtained. Such a process guarantees a minimum disturbance for the raw data and avoids non-necessary information loss due to spectra preprocessing.

### Machine Learning and Deep Learning Settings

For data dimension reduction and visualization, we implemented principal component analysis (PCA) via scikit-learn version 1.3.2.^31^ The CoVari deep learning algorithm was developed for spectrum classification and regression using TensorFlow version 2.15.0. It had two output heads to simultaneously process the classification of the virus type and quantify the virus concentration (step 10). All implementations were coded in Python 3.11.0. and run on a desktop with an Intel i7-13700KF CPU @ 3.40 GHz, 64GB of RAM, and an NVIDIA GeForce RTX 4080 GPU. Note that the specific architecture and training parameters of the CoVari model were carefully selected and are detailed in the corresponding sections for clarity.

## Results and Discussion

### ACE2-SERS Sensor Characterization

**Figure 2:**
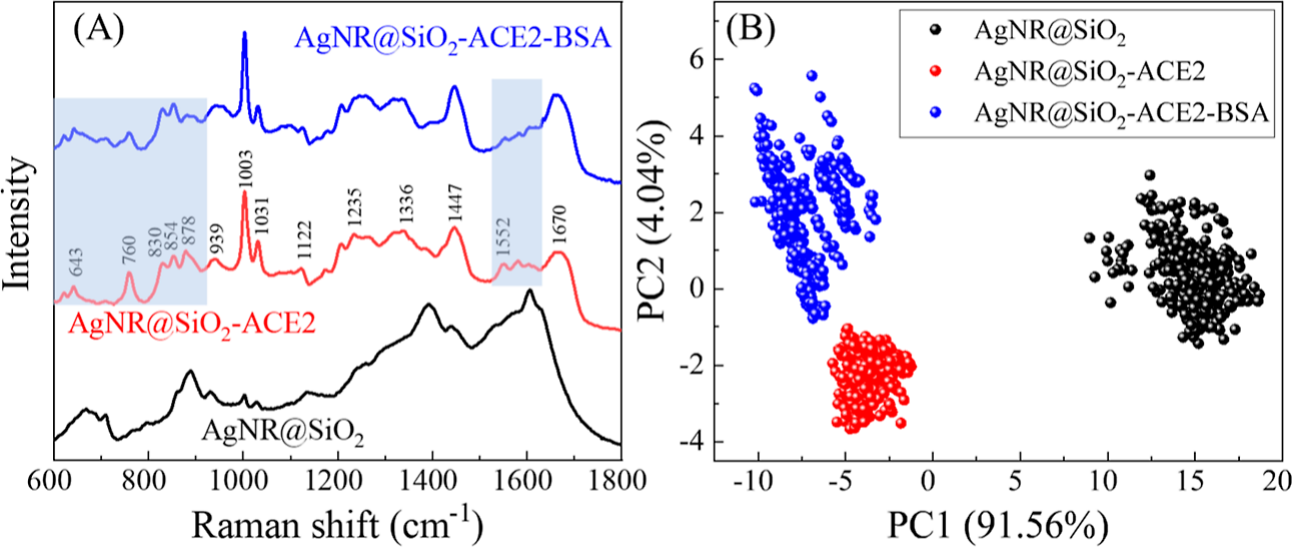
(A) SERS spectra of the AgNR@SiO_2_ substrate, AgNR@SiO_2_-ACE2-, and BSA-treated AgNR@SiO_2_-ACE2. (B) Corresponding PCA plot.

Figure 2A illustrates sequential SERS spectra captured during the ACE2 immobilization and BSA blocking processes. The untreated AgNR@SiO_2_ substrate displays some background SERS peaks (depicted by the black curve). Following the ACE2 immobilization, distinct SERS characteristic peaks of ACE2 emerge, as indicated in the red spectrum. The specific peak assignments for the ACE2 protein are outlined in Table S2. The optimal ACE2 immobilization condition, determined to be a 124 μg/mL concentration, follows methods outlined in a previous report.^32^ After BSA blocking, the SERS spectrum (represented by the blue curve) closely resembles the ACE2 spectrum, with slight alterations observed in the wavenumber ranges of Δ*v* < 900 cm^–1^ and Δ*v* ∼ 1500–1650 cm^–1^ (refer to the shaded areas in Figure 2A). Upon applying a simple chemometric technique, PCA, to analyze these consecutive spectra (as depicted in Figure 2B), it is evident that spectra from each step form a distinct, well-separated cluster. This outcome serves as confirmation of the successful ACE2 immobilization and BSA blocking process.

### Specificity Test of the SERS Sensor

**Figure 3:**
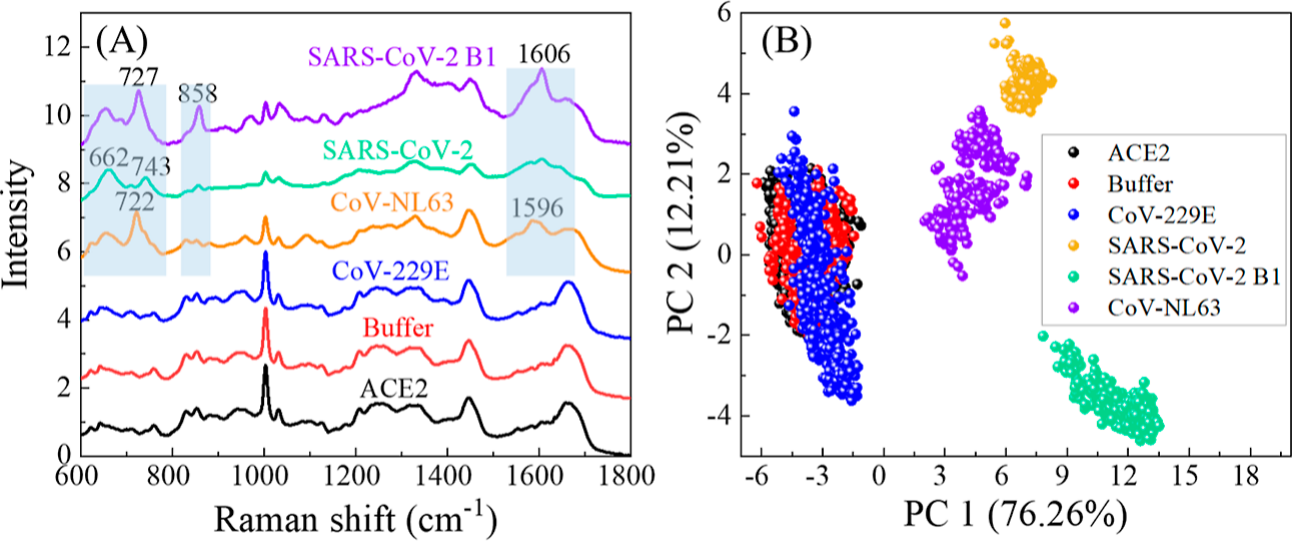
(A) Average SERS spectra of the ACE2-modified SERS sensor (ACE2), buffer, CoV-229E (10^5^ PFU/mL), CoV-NL63 (10^5^ PFU/mL), SARS-CoV-2 (10^5^ PFU/mL), and SARS-CoV-2 B1 (10^5^ PFU/mL). (B) Corresponding PCA plot, i.e., Principal Component 2 (PC2) versus Principal Component 1 (PC1) plot.

To assess the specificity of the SERS sensor, five different specimens were prepared: buffer and CoV-229E (10^5^ PFU/mL) were used as references because CoV-229E cannot be specifically captured by ACE2. On the other hand, CoV-NL63, SARS-CoV-2, and SARS-CoV-2 B1 (all at 10^5^ PFU/mL), which all can be captured by ACE2, were employed as target analytes. The results are summarized in Figure 3.

In the SERS spectra of buffer and CoV-229E (red and blue curves in Figure 3A), no significant deviations were noted compared to the ACE2 spectra. Contrastingly, for CoV-NL63 (orange curve), SARS-CoV-2 (green curve), and SARS-CoV-2 B1 (purple curve), distinguished changes in SERS spectra are observed in the wavenumber regions of Δ*v* ∼ 600–777, 817–877, and 1521–1649 cm^–1^ (highlighted in light blue shading), particularly noticeable peaks at Δ*v* = 727, 858, and 1606 cm^–1^. Notable differences are also observed within these regions among the three spectra. For instance, in the Δ*v* ∼ 817–877 cm^–1^ region, SARS-CoV-2 B1 exhibits a sharp peak at Δ*v* = 858 cm^–1^, whereas CoV-NL63 and SARS-CoV-2 displays two less pronounced peaks, similar to those observed in the reference group. In the Δ*v* ∼ 1521–1649 cm^–1^ region, SARS-CoV-2 B1 has a distinct and relatively sharp peak at Δ*v* = 1606 cm^–1^. In contrast, the corresponding peak in the CoV-NL63 spectrum is less pronounced, and SARS-CoV-2 exhibits a broader peak. Other differences can also be visually identified in other wavenumber regions. These differences, observed across various wavenumber regions, serve as the basis for differentiation among the spectra and distinguish them from the references. PCA was conducted using the spectra of the five analytes and the ACE2 background depicted in Figure 3A to illustrate the differences among these spectra better. The resulting clusters, shown in Figure 3B by plotting the scores of PC2 against PC1, reveal significant insights. The combined variance explained by PC1 and PC2 approximates 88%, indicating that these two PCs effectively encapsulate the data set’s information. This robust representation allows for a reliable interpretation of the subsequent findings. It is evident that the clusters corresponding to buffer (red) and CoV-229 (blue) overlap and intermingle with the ACE2 cluster (black), suggesting similarity in the spectra from these three cases. In contrast, the clusters associated with CoV-NL63 (orange), SARS-CoV-2 (green), and SARS-CoV-2 B1 (purple) are not only distinctly separated from the reference clusters but also segregated from each other. This outcome demonstrates the impressive efficacy of the proposed SERS detection approach in distinguishing target analytes from references and in differentiating distinct SARS-CoV-2 variants. In essence, the SERS spectra show high specificity for distinguishing various SARS-CoV-2 variants.

### Concentration-Dependent Virus Detection

**Figure 4:**
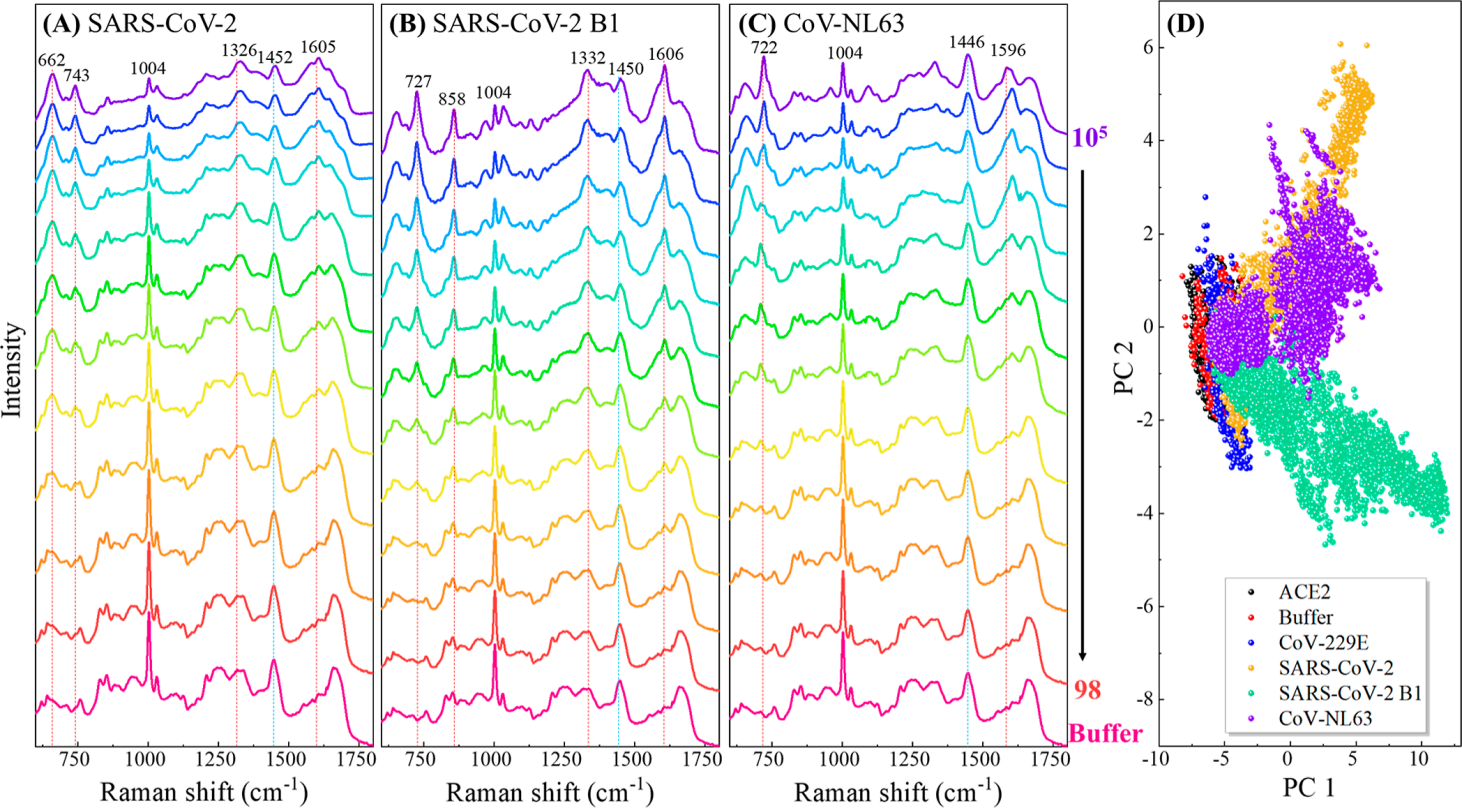
Average SERS spectra of (A) SARS-CoV-2, (B) SARS-coV-2 B1, and (C) CoV-NL63 at different concentrations. The concentrations are labeled beside the SERS spectra with the unit of PFU/mL. (D) PCA plot for SERS spectra of three viruses at different concentrations as well as references (ACE2, Buffer, and CoV-229E).

The above results clearly demonstrate the proficiency of the ACE2-based SERS sensor in detecting SARS-CoV-2. To further assess its detection capability, concentration-dependent measurements were conducted. Figure 4 shows the average SERS spectra for various concentrations of SARS-CoV-2, SARS-CoV-2 B1, and CoV-NL63. All the SERS spectra exhibit distinct peaks at Δ*v* = 1004 and 1452 cm^–1^ (or nearby peaks at 1450 or 1446 cm^–1^, indicated by the blue dashed lines in Figure 4A–C), due to ACE2. At the highest concentration of SARS-CoV-2 (10^5^ PFU/mL), new characteristic peaks at Δ*v* = 662, 743, and 1605 cm^–1^ emerge, as indicated by the red dashed lines in Figure 4A. As the SARS-CoV-2 concentration decreases, the relative intensities of these peaks decrease. At the same time, the Δ*v* = 1004 and 1452 cm^–1^ peaks become more prominent, i.e., the SERS spectrum gradually resembles that of ACE2 or buffer, highlighting the progressive spectral change with decreasing viral concentration. For SARS-CoV-2 B1 at 10^5^ PFU/mL, unique peaks at Δ*v* = 727, 858, 1332, and 1606 cm^–1^, specific to SARS-CoV-2 B1, are observed (see Figure 4B). Similarly, for CoV-NL63 at 10^5^ PFU/mL, distinct peaks at Δ*v* = 722 and 1596 cm^–1^ appear (see Figure 4C). With lower viral concentrations, the relative intensities of these peaks gradually diminish. Thus, at lower viral concentrations, distinguishing these spectra becomes challenging due to interference from the ACE2 spectrum, even with the application of chemometric methods. Figure 4D presents the PCA plot for the spectra of all concentrations. Clusters for the same viral type but with different concentrations heavily overlap, illustrating the difficulty of utilizing classic chemometric methods to differentiate viral types based solely on SERS spectra. Moreover, predicting the virus suspension’s concentration using conventional regression methods (e.g., linear fitting for SERS peak intensities versus concentrations) becomes even more challenging. Consequently, a deep learning strategy is developed to enhance the differentiation and quantification of viral infections.

### Deep Learning Model to Classify and Quantify the SERS Spectra

**Figure 5:**
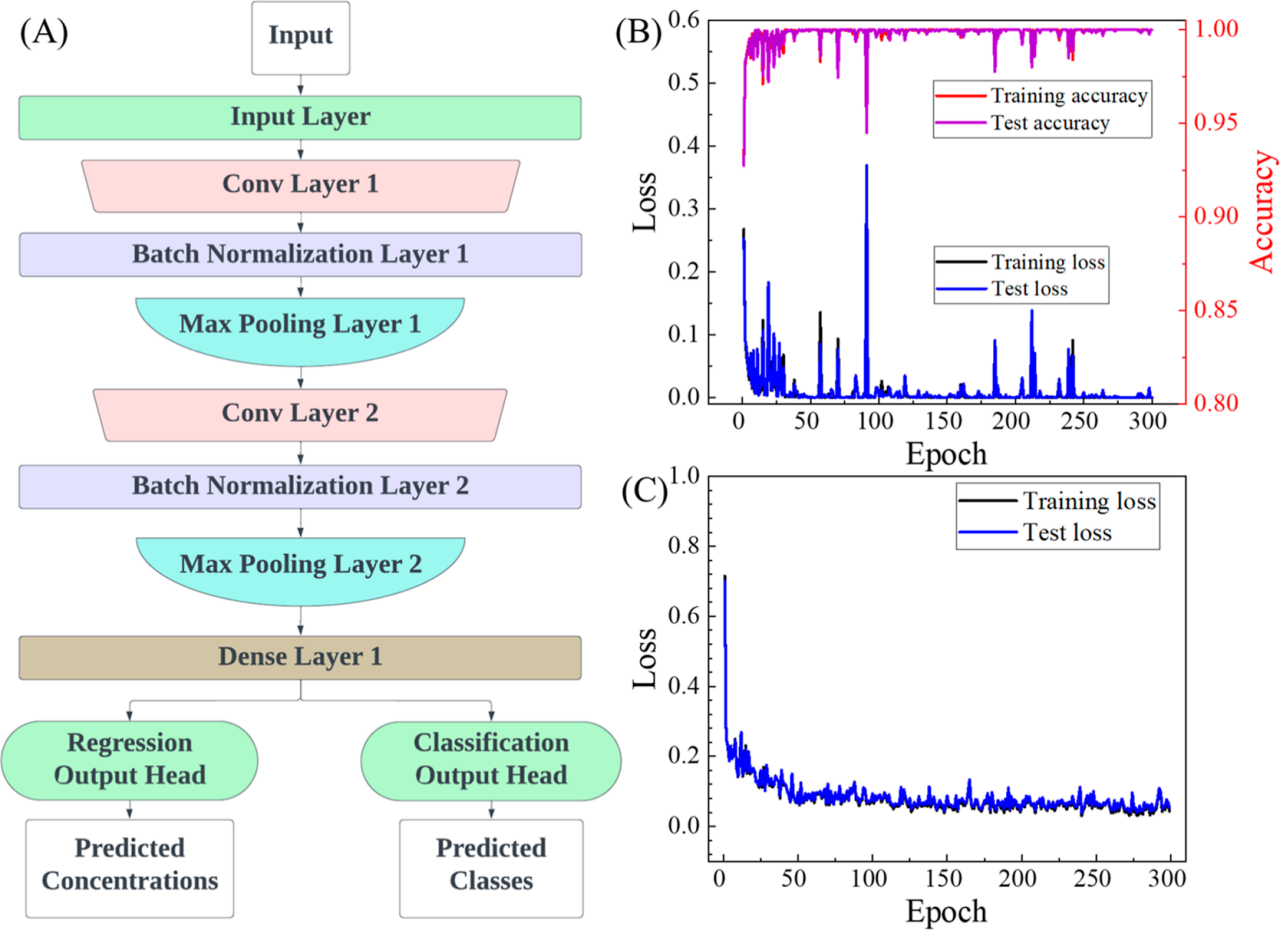
(A) Architecture of the CoVari algorithm. The plots of (B) classification loss and accuracy versus training epoch and (C) regression loss during the CoVari’s training and testing.

A deep learning model called CoVari was developed to classify the virus types simultaneously and predict the viral concentrations based on the SERS spectra. CoVari contains convolutional neural networks (CNNs). CNNs are feedforward neural networks that can efficiently process spatially hierarchical data, such as spectra. They utilize local receptive fields, shared weights, and pooling layers.^33^ The local receptive fields enable CNNs to focus on small regions of the input space, capturing local patterns effectively. Shared weights reduce the model’s complexity and the number of parameters compared to fully connected networks by reusing the same weights across different parts of the input, thereby facilitating the detection of similar features in different regions. Pooling layers contribute to dimensionality reduction by downsampling their input, further helping to extract and emphasize essential features while also providing a form of translation invariance. Together, these characteristics enable CNNs to reduce dimensionality and extract salient features from input data.^34^ The detailed architecture of the CoVari deep learning algorithm is shown in Figure 5A. It consists of two convolutional layers, each followed by a batch normalization (BN) layer and a maximum pooling layer. Both convolutional layers incorporate 64 filters of size 3 × 3 and utilize the rectified linear unit (ReLU) activation function. These convolutional layers play a crucial role in detecting and learning intricate patterns and features of the input data. Following each convolutional layer is a BN layer, which normalizes, recenters, and rescales the data, enhancing the efficiency and stability of the network’s training process. After the BN layers are three 1D max-pooling layers, each with a pooling kernel size of 8 and a stride length of 2. These layers serve to down-sample the output of the BN layers by selecting the maximum value within each window, effectively reducing the dimensionality of the input spectra. The network then transitions to a fully connected (dense) layer, which condenses the dimensions to 200. Following this, two output heads operate in parallel to predict the virus’s type and concentration. The final output of the CoVari deep learning algorithm for a given SERS spectrum consists of two four-element vectors, denoted as [*V*_1_, *V*_2_, *V*_3_, *V*_4_], [*C*_1_, *C*_2_, *C*_3_, *C*_4_]. The first four elements, *V*_1_ to *V*_4_, with *V*_*i*_ = 0 or 1, indicate the virus type: *i* = 1 for SARS-CoV-2, *i* = 2 for SARS-CoV-2 B1, *i* = 3 for CoV-NL63, and *i* = 4 for reference, respectively. The second four elements, *C*_1_ to *C*_4_, represent the corresponding log_10_ concentration of *V*_1_ to *V*_4_. To handle reference data where the concentration should be zero on a linear scale but would result in negative infinity on a logarithmic scale, the concentration of reference samples was deliberately set to −20 in logarithmic form. This value is sufficiently different from the viral concentration range (logarithmic scale of 2–5) to ensure proper training of the model. For example, the vectors [0, 1, 0, 0], [0, 5, 0, 0] represent the prediction of the presence of SARS-CoV-2 B1 at a concentration of 10^5^ PFU/mL; and [0, 0, 0, 1], [0, 0, 0, −20] represent the prediction of the presence of no viruses but only reference. It is important to note that only the concentration corresponding to the identified virus type should be considered when interpreting the output vector. Here, the SERS spectra from ACE2, buffer, and CoV-229E are attributed as references. A custom loss function is defined for both classification and regression. For classification, we compute the cross-entropy based on the predicted virus types [*V*_1_, *V*_2_, *V*_3_, *V*_4_] against the actual virus types, while for regression, we use the MAE only for the concentration prediction at the predicted virus type. The overall loss function is a linear combination of these two terms,
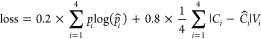
1

The first term in eq 1 is the cross-entropy, where *p*_*i*_ and  are the true and predicted probabilities of *V*_*i*_ type; the second term results from regression, where *C*_*i*_ and  are the true virus concentration and predicted virus concentrations, respectively. This loss function ensures that the model focuses only on relevant predictions, optimizing computational resources by not calculating unnecessary concentrations. In addition to the previously mentioned use of the ReLU activation, other settings for this CoVari deep learning algorithm include the use of Adam optimizer with a learning rate of 0.001, a total of 300 training epochs with a batch size of 32. According to the properties of the CoVari deep learning algorithm’s architecture and the output vector, this algorithm can be generalized to simultaneously predict viral variant species and concentrations.

Table S1 shows the number of SERS spectra from references and viruses at various concentrations. To assess the robustness and reproducibility of the CoVari, we performed a 10-fold cross-validation. The entire spectra data set was divided into 10 subsets, or folds, employing stratified sampling to ensure a representative distribution across all sets. Then, each subset was used as the test set once, while the remaining nine served as the training set to train a model with the architecture described in Figure 5A. During the training and validation of each fold, changes in classification loss and accuracy over epochs (up to 300 epochs) were monitored. Here, accuracy is defined as the ratio of correctly predicted instances to the total predictions made by the model. Overall, all 10 cross-validations have quickly achieved convergence for classification within 150 epochs and regression within 300 epochs. The representative curves of classification loss and accuracy over epochs of this model are shown in Figure 5B. Similarly, the regression loss is plotted in Figure 5C. The proposed model achieves an average accuracy of 99.9 ± 0.1% in discriminating between different virus variants and *R*^2^ values larger than 0.98 for quantifying viral concentrations of the three viruses of SARS-CoV-2, SARS-CoV-2 B1, and CoV-NL63, demonstrating the high quality of the detection. Figure S2 summarizes the overall performance of 10-fold cross-validations. These results demonstrate that the CoVari model is accurate and fast in training. The model’s shallow architecture, comprising just two convolutional layers, further contributes to its rapid inference capabilities. The entire training process for 300 epochs was completed in approximately 15 min. The inference time for evaluating the test spectra for each fold (around 1200 spectra) was within 1 s.

**Figure 6:**
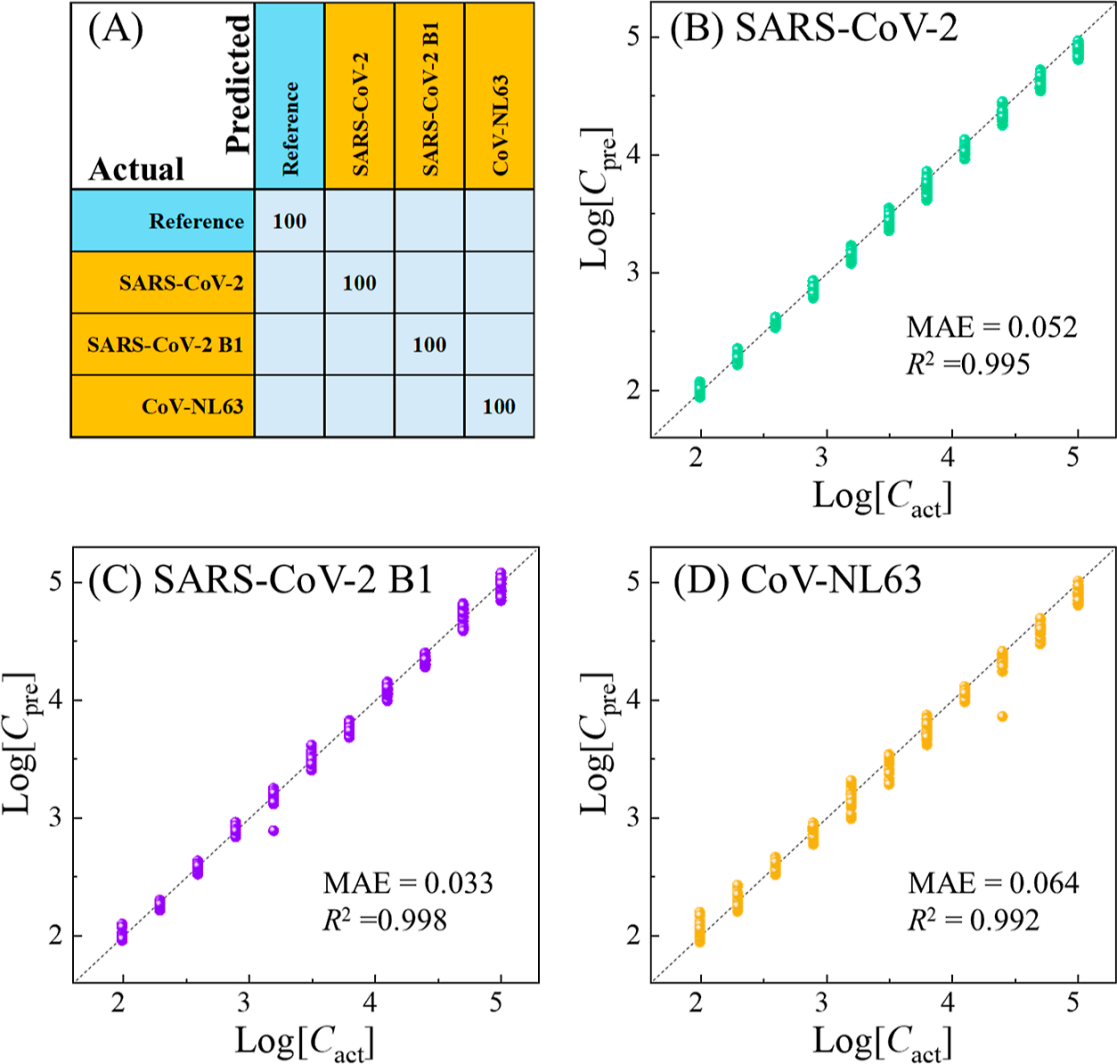
(A) Confusion matrix of the CoVari for detecting three virus specimens and references. The regression results of the CoVari for (B) SARS-CoV-2, (C) SARS-CoV-2 B1, and (D) CoV-NL63. The *x*-axis is log_10_(*C*_act_) of testing spectra, and the *y*-axis is log_10_(*C*_pre_). The dashed lines represent log_10_(*C*_act_) = log_10_(*C*_pre_). The unit of the concentrations is PFU/mL.

Figure 6A plots the confusion matrix from a representative cross-validation, where an impressive 100% accuracy is obtained for the overall prediction of various virus species and variants using SERS spectra from different concentrations. Figure 6B–D shows the regression results, where the predicted concentration (*C*_pre_) of viruses from the CoVari and actual concentration (*C*_act_) are plotted in the log–log scale. These log_10_(*C*_pre_) – log_10_(*C*_act_) data align closely with the linear relationship log_10_(*C*_pre_) = log_10_(*C*_act_), as indicated by the dashed diagonal lines. The variation of the predicted concentrations is consistently small across different *C*_act_ values, demonstrating the model’s precision across all concentrations. Linear fitting of these data points yields coefficients of determination (*R*^2^) values of 0.995, 0.998, and 0.992 for SARS-CoV-2, SARS-CoV-2 B1, and CoV-NL63, respectively. These results show that the proposed SERS + CoVari deep learning algorithm based on the ACE2-modified SERS substrate can achieve high classification accuracy and precise quantification performance for different coronavirus species.

**Figure 7:**
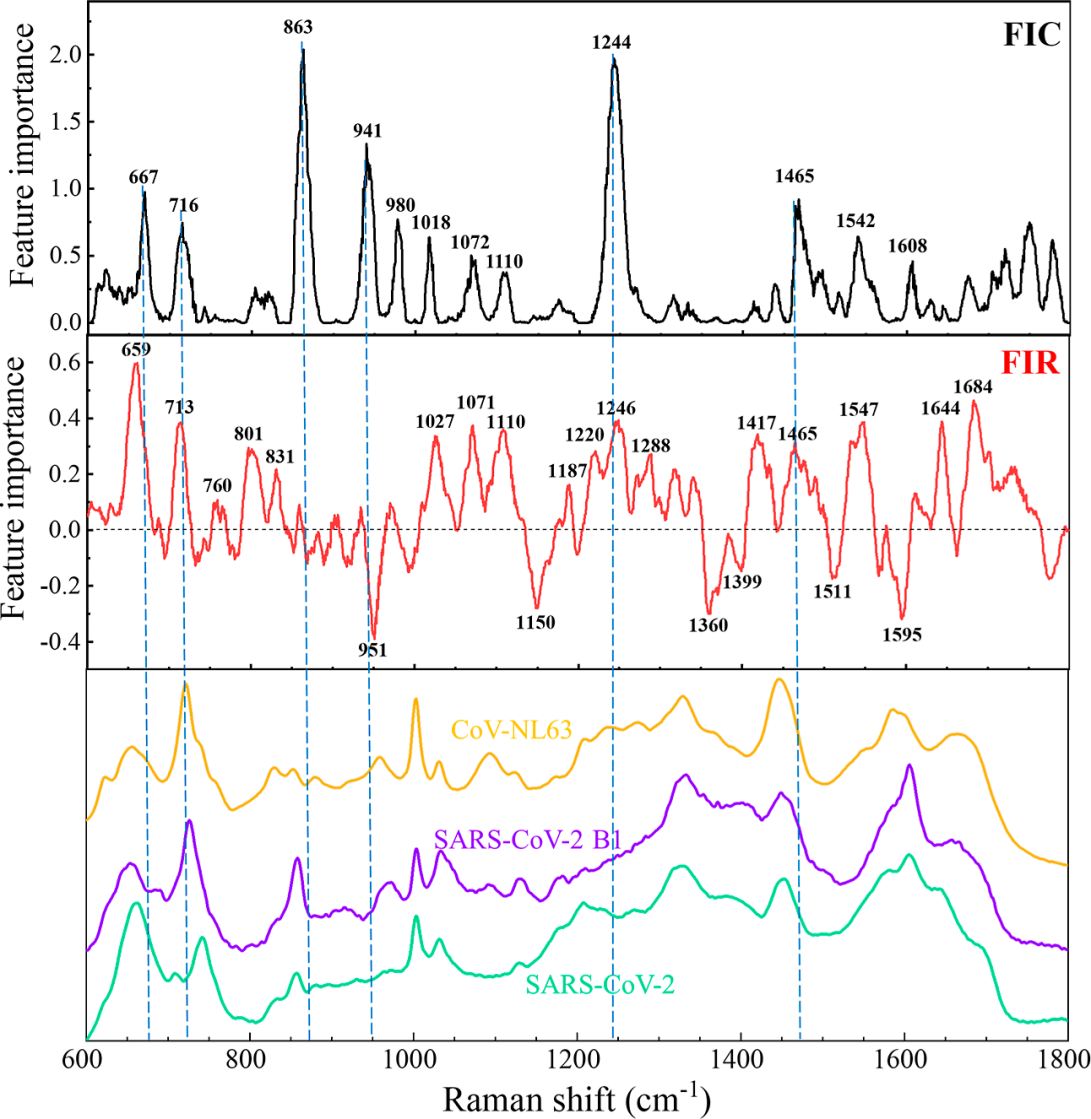
Feature importance plots for classification and regression, and comparison of SERS spectra of three viruses.

To better understand the results from the CoVari algorithm, we calculated the feature importance of classification (FIC) and the feature importance of regression (FIR) based on a permutation algorithm;^35^ detail is described in Section S3 of Supporting Information. Figure 7 plots the resulting FIC (black) and FIR (red), alongside a comparative analysis with the SERS spectra of three different coronaviruses at 10^5^ PFU/mL concentrations. The prominent important feature peaks for FIC are at Δ*v* = 863, 941, 1244, and 1465 cm^–1^, which can be attributed to vibrations of Tyr (β-sheet), N–C_α_–C, Amide III, and C–H (def), respectively. Such peaks in the FIC are significant, as they highlight the distinct spectral differences among the three viruses, i.e., CoVari relies on these peaks to classify viruses. Other peak assignments for FIC are listed in Table S3. It is noted that the peaks in the FIC do not align with SERS peaks of viruses, but some peaks in the FIC indicate the slopes (first derivative) of the spectra are different, e.g., at Δ*v* = 863 and 1244 cm^–1^. On the contrary, the shared peaks at Δ*v* = 1004 and 1448 cm^–1^, although present in the spectra, do not contribute significantly to the differentiation between the virus types.

Compared to FIC, FIR shows more feature peaks, e.g., at Δ*v* = 659, 713, 951, 1071, 1150, 1246, 1360, 1547, and 1595 cm^–1^, along with many other small peaks. Peak assignments for the feature importance of the regression are listed in Table S3. However, most feature peaks are intriguingly different from the distinct SERS spectral peaks associated with the three coronaviruses. In addition, the FIC shows negative values, which means that removing some features (spectra wavenumber regions) improves the regression results according to the definition of  shown in Section S3 of Supporting Information. This indicates that performing feature selection before model training may lead to a better model for virus detection, which will be a future research scope. In comparison, FIR has more features than FIC, and the increased complexity in FIR arises due to the following reasons: (1) nature of output: classification aims to assign data points to predefined viral categories. In contrast, regression involves predicting continuous numerical values, which can exhibit a wider range of variations compared to the discrete categories in classification. This requires considering more features to accurately model the intricate relationships between input spectra and output of viral concentrations. (2) Sensitivity to spectra details: small variations in input spectral features can lead to substantial changes in viral concentration predictions in regression. As demonstrated in Figure 4A–C, spectra belonging to the same virus type can exhibit drastic changes in concentration even with minimal shifts in intensity magnitude. This sensitivity requires a more comprehensive consideration of spectral features that might have relatively less impact in classification scenarios. (3) Range of outputs: regression deals with a broader range of potential outputs, specifically a continuous range spanning approximately 10^2^ to 10^5^ in this context. This necessitates the identification and analysis of nuanced relationships between spectral features and the resulting viral concentrations. This broader range increases the likelihood of needing more features to adequately account for the diverse possibilities within the output space. (4) Incorporating complex relationships: regression models may need to capture complicated interactions and nonlinearities between spectral features and output viral concentrations, leading to more comprehensive feature consideration.

### LOD Determination

The LOD is a critical parameter in spectroscopic analysis, particularly when combined with machine learning techniques for accurately identifying and quantifying pathogens. We determined the LOD using the following formulas^36^
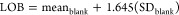
2
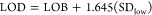
3where the limit of blank is calculated as the mean of the predicted concentration of the reference samples (mean_blank_), plus 1.645 times the standard deviation of the predicted concentration of these reference samples (SD_blank_). The multiplier 1.645 corresponds to ta normal distribution’s 95% confidence interval, indicating that 95% of reference samples should be statistically distinguishable from those with low concentrations. Subsequently, the LOD is determined by adding 1.645 times the standard deviation of the predicted samples with low viral concentrations (SD_low_). In this study, these “low concentration samples” are defined as those with actual concentrations of 98 PFU/mL, the lowest possible concentration. According to the results from the CoVari deep learning algorithm, the LOD for SARS-CoV-2, SARS-CoV-2 B1, and CoV-NL63 are calculated to be 10.47, 11.88, and 21.59 PFU/mL, respectively. These values provide a benchmark for the sensitivity of our SERS-based detection system, offering a comparative perspective against traditional methods and existing literature. Detailed calculation process values can be found in Section S4 of Supporting Information. For comparison, traditional calibration curves for three coronaviruses are also constructed, as shown in Section S5 of Supporting Information. Still, the SERS peak intensities do not show a distinguishable change at low virus concentrations. According to the definition of the LOD from eqs 2 and 3, the LODs for SARS-CoV-2, SARS-CoV-2 B1, and CoV-NL63 are estimated to be 991, 1513, and 13,335 PFU/mL, respectively, based on the traditional calibration curves. These values are significantly higher than those determined by CoVari.

### Unknown Specimen Test

**Figure 8:**
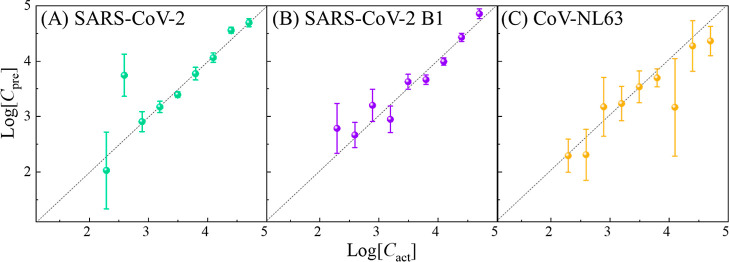
(A) Regression results of the CoVari for detection of three viruses with unknown concentrations in buffer for (B) SARS-CoV-2, (C) SARS-CoV-2 B1, and (D) CoV-NL63. The *x*-axis is log_10_(*C*_act_) of testing spectra, and *y*-axis is log_10_(*C*_pre_). The dashed lines represent log_10_(*C*_act_) = log_10_(*C*_pre_). The unit of the concentrations is PFU/mL.

**Figure 9:**
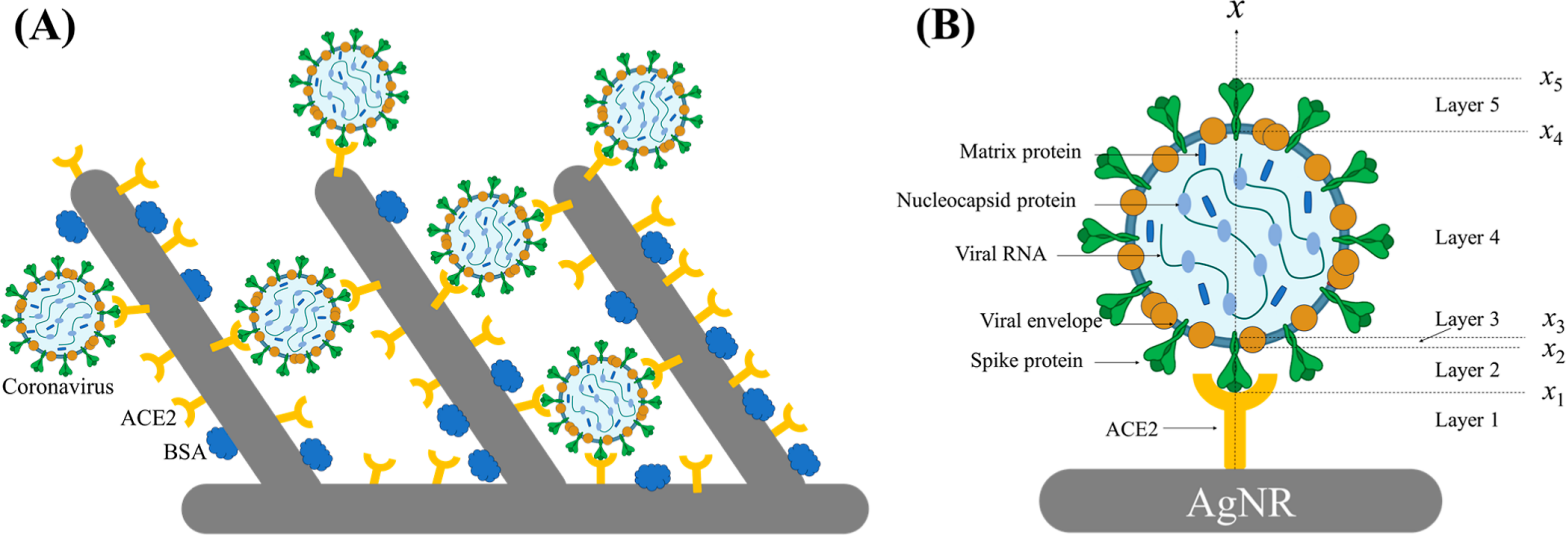
(A) Diagram of virus detection using ACE2-modified AgNR substrates. (B) Layered structure of a virus attached to one Ag nanorod.

Practically, when a patient is scheduled for a viral infection diagnostic test, the virus’s type and concentration in the specimen are unknown. While it can be assumed that the virus belongs to a predefined set recognizable by the model, the actual concentration in real-world cases may not align with those used in the training sets. Consequently, it is crucial to evaluate the performance of the CoVari deep learning algorithm using SERS spectra obtained from samples with concentrations that were not included in the training phase, thereby ensuring the model’s robustness and accuracy in real-life diagnostic applications. To mimic this situation, the following procedure was designed: from the 33 sets of SERS spectra with different viruses and concentrations in Table S1, 3 sets of SERS spectra from three viruses at the same concentration (for example, 50,000 PFU/mL) were taken out from the training set and only used as the testing spectral set. The rest of the 30 sets of SERS spectra were used as the training spectral sets to establish the CoVari deep learning algorithm, i.e., the training spectral sets did not contain any spectra from the testing spectral set. This approach was iteratively applied for each virus, repeating the process 9 times to account for the 9 distinct concentration levels available (excluding the highest and lowest concentration sets for each virus, which were set aside for further evaluation as they define the concentration boundaries). During these procedures, both classification and quantification were carried out. The classification accuracies are summarized in Table S5. The classification gives more than 90% accuracy for most viruses with different concentrations, except for CoV-NL63 at 195, 391, and 781 PFU/mL and SARS-CoV-2 at 195 PFU/mL. The regression results are shown in Figures 8 and S5. All the predicted concentrations *C*_pre_ align with the actual concentration *C*_act_ with MAEs of 0.2912, 0.2358, and 0.3941 for SARS-CoV-2, SARS-CoV-2 B1, and CoV-NL63, respectively. Lower concentrations of SARS-CoV-2 and SARS-CoV-2 B1 exhibit larger variations. Compared to the corresponding values obtained in Figure 6, the MAEs in unknown concentration predictions are generally larger, and *R*^2^ values are lower, especially for CoV-NL63. This can be attributed to several factors. First, the CoVari deep learning algorithm already contains spectral data sets from all concentrations for the known concentration regression, enabling it to predict the testing spectra based on known familiar patterns. In contrast, for the unknown concentration regression, the CoVari deep learning algorithm needs to use interpolation to obtain the result, which requires the model to predict the concentration beyond its trained range. Interpolation is inherently more challenging due to unseen relationships and potential outliers, leading to increased prediction errors. Second, the training data might insufficiently represent the diverse range of possible concentrations. The CoVari deep learning algorithm may not have learned the complex relationships necessary to predict all possible concentrations accurately. The presence of outliers or anomalies in the SERS spectra of unknown concentrations can further confound predictions, as the model lacks prior exposure to such instances. Moreover, the complexity of underlying processes for unknown concentrations might surpass the model’s capacity to capture them from limited training spectra. Overall, the larger MAEs and smaller *R*^2^ in unknown concentration predictions arise due to the difficulties of interpolation, limited representational diversity of known data, the presence of outliers, different variations between training and unknown concentration, and the complexity of underlying relationships. In principle, the SERS spectra obtained from this virus detection strategy may be written as the linear combination of the following contributions according to Figure 9A
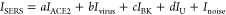
4where *I*_ACE2_ is the SERS signal from ACE2 protein, *I*_virus_ is the SERS spectrum due to the virus/spike-protein binding, *I*_BK_ is the SERS signal from background, e.g., BSA proteins, *I*_noise_ is the electronic noise inherent to the Raman instrument, independent of the instrument’s optical response, *I*_U_ is some unknown components or contaminations, and *a*–*d* are the coefficients for the corresponding SERS signals. For *I*_virus_, we can treat the virus/spike protein binding to ACE2 on the AgNR as the layered structure, as shown in Figure 9B:^37^ the first layer consists of ACE2 and BSA proteins; the second layer consists of spike proteins; the third layer is the viral envelope (lipid bilayer); the fourth or fifth layer comprises of the matrix protein, nucleocapsid proteins, viral RNA, and spike protein;^38^ thus, *I*_virus_ can be written in an integration form. Thus, the SERS intensity can be expressed as
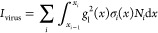
5where *g*_l_(*x*) is the location-dependent local field enhancement factor along the *x*-direction, σ_*i*_(*x*) is the combined SERS scattering cross-section of molecules in the *i*th layer, and *N*_*i*_ is the effective number of the virus component molecules in the *i*th layer. Note that *g*_l_(*x*) decays along the *x*-direction. It is expected that the coefficients *a*–*d* are significantly influenced by the concentration of virus (and spike proteins), and the overall spectral shape of *I*_SERS_ as well as *I*_ACE2_, *I*_virus_, and *I*_BK_ or *I*_U_ will be altered by the concentration of virus (and spike proteins). Especially at a low concentration, viruses cover only a small portion of the surface, the virus–ACE2 binding may change the orientation of ACE2 molecules, resulting in different virus orientations. The virus and other components in the solution will compete with the adsorption on the AgNR substrates, which could alter the amount of contaminants in the hotspot of the SERS substrates. These effects not only change the coefficients but also altered the spectral shapes of *I*_ACE2_, *I*_Virus_, *I*_BK_, and *I*_U_. Such changes indicate a strong nonlinear relationship in the low-concentration region. In addition, *I*_noise_ is sufficiently strong compared to the change in SERS spectrum after low-concentration virus binding, resulting in a low signal-to-noise ratio and bringing a large variation in spectra. Thus, without including sufficient SERS spectra from low concentrations in the training spectral set to accurately predict corresponding concentrations in this concentration region becomes challenging. So, larger error/variance can be observed in unknown concentration tests. To further improve the model performance, several methods can be attempted for future research directions, such as increasing model complexity, enhancing models to handle interpolation better, and integrating uncertainty quantification of *I*_U_ into predictions, etc. Despite these challenges, the overall predicted virus concentrations align well with the trend of the actual concentration, demonstrating the effectiveness of the proposed framework for virus quantification. Despite these challenges, the overall predicted concentration does match well with the actual concentration. The average predicted concentrations do not deviate by more than 1 order of magnitude from the actual concentrations (Figure 8). This consistent alignment demonstrates that the proposed SERS + CoVari deep learning algorithm is effective for virus variant detection and quantification.

### SARS-CoV Spike Protein Detection in Saliva

To further assess the detection capability in a real-world environment of the proposed strategy, saliva specimens were selected as a complex matrix because it offers a noninvasive and easily collectible alternative, gaining attention in recent years.^39,40^ Due to the limited access to the BSL-3 lab, three coronavirus spike protein variants were selected for the demonstration, including SARS-CoV-2 spike, SARS-CoV-2 spike (BA 2.75.2), and SARS-CoV-1 spike. Concentration-dependent measurements of three spike proteins ranged from 25.6 pg/mL to 50 μg/mL in saliva. Figure S6 shows the average SERS spectra for various concentrations of three coronavirus spike proteins. All the SERS spectra from three spike proteins are very similar to those from reference (saliva) or ACE2-modified SERS substrates themselves, exhibiting distinct peaks at Δ*v* = 1004 and 1452 cm^–1^, which indicates that the capture of spike proteins will not induce a significant change in the SERS spectrum, even for the spectra from high-concentration specimens. There are only minor visual changes in the spectra region from 1550 to 1620 cm^–1^ marked by the dashed boxes. For the training of the CoVari, SERS spectra were randomly chosen from Table S6 for 10-fold cross-validation with stratified sampling and a similar sampling strategy with virus detection. The CoVari still shows good performance on spike protein classification and quantification. All 10-fold cross-validations achieve convergence for classification within 250 epochs and regression within 300 epochs. The representative curves of classification loss and accuracy over epochs of this model are shown in Figure S7A, and the regression loss is plotted in Figure S7B. The proposed CoVari achieves an average accuracy of 99.9 ± 0.1% in discriminating between different coronavirus spike protein variants and *R*^2^ values larger than 0.98 for quantifying concentrations of SARS-CoV-2 spike, SARS-CoV-2 spike (BA 2.75.2), and SARS-CoV-1 spike, demonstrating high-quality detection in saliva. Figure S8 summarizes the detailed performance metrics obtained from 10-fold cross-validation. They show similar regression results with small MAEs < 0.2, large *R*^2^ > 0.99, and low LODs ∼ 10^–11^ g/mL for all three spike proteins. Figure S9A plots a representative confusion matrix, showing 100% classification accuracy for the three coronavirus spike proteins at different concentrations using SERS spectra. Figure S9B–D shows the corresponding regression results, where the predicted concentration (*C*_pre_) of spike proteins from CoVari against the actual concentration (*C*_act_) is plotted in a log–log scale. These log_10_(*C*_pre_) – log_10_(*C*_act_) data align closely with the linear relationship log_10_(*C*_pre_) = log_10_(*C*_act_), as indicated by the dashed diagonal lines. Linear fitting of these data points yields coefficients of determination (*R*^2^) values of 0.993, 0.996, and 0.996 for SARS-CoV-2 spike, SARS-CoV-2 spike (BA 2.75.2), and SARS-CoV-1 spike, respectively. These results demonstrate that the proposed SERS sensor combined with CoVari has the potential to achieve high classification accuracy and precise quantification performance for different coronavirus species/variants in real-world environments.

## Conclusions

In summary, an ACE2-functionalized AgNR@SiO_2_ SERS sensor is developed for three SARS-CoV-2 variant classifications and quantifications. By combining SERS measurements and the CoVari deep learning algorithm, this detection strategy can accurately detect and quantify coronaviruses and their variants. By applying 10-fold cross-validation, the proposed method demonstrates exceptional detection quality by achieving an average of 99.9% accuracy rate in distinguishing between different virus species, with *R*^2^ values of approximately 0.993 for SARS-COV-2, 0.996 for SARS-COV-2 B1, and about 0.988 for COV-NL63. In tests with unknown concentrations, the classification accuracy remains larger than 90% for most cases, especially for concentrations greater than 781 PFU/mL. The predicted concentrations consistently align with actual values, highlighting the robustness of the CoVari deep learning algorithm in both classification and quantification tasks. The LOD of the sensor is determined to be 10.472, 11.882, and 21.591 PFU/mL for SARS-CoV-2, SARS-CoV-2 B1, and CoV-NL63, respectively. Exploiting the properties embedded in the architecture of CoVari deep learning algorithms, along with the characteristics of the output vector, this versatile algorithm can be extended to predict viral variant species and their concentrations simultaneously across a diverse spectrum of virus variants. The algorithm’s notable advantages include its adaptability to evolving virus landscapes, robust generalization capabilities, and efficient handling of complex data sets. Furthermore, its inherent scalability facilitates the incorporation of new SERS spectra, ensuring continuous improvement in predictive accuracy. These results demonstrate that a combination of SERS and the CoVari model can serve as a valuable tool for comprehensive and real-time monitoring of viral dynamics, potentially enhancing medical diagnosis and facilitating therapeutic interventions.

## Data Availability

The data and codes supporting the main findings of this study are available from the corresponding authors upon reasonable request.

## Supporting Information Available

Characterization of the SERS substrate, additional information for coronavirus detection, calculation of the permutation feature importance, calculation of the LOD, traditional calibration curves for coronavirus detection, additional results for the unknown specimen test, and additional information on spike protein detection in saliva (PDF)

## Notes

During the preparation of this work the authors used ChatGPT in order to proof-write the manuscript, i.e., to get rid of possible grammar errors and change the sentence structures. After using this tool/service, the authors reviewed and edited the content as needed and take full responsibility for the content of the publication.
