# Supplementary material for: Rapid Detection of SARS-CoV-2 Variants Using an Angiotensin-Converting Enzyme 2-Based Surface-Enhanced Raman Spectroscopy Sensor Enhanced by CoVari Deep Learning Algorithms
Source: ACS Sens. 2024 Jun 6;9(6):3158–69. doi: 10.1021/acssensors.4c00488 (PMC11217934; doi:10.1021/acssensors.4c00488)
Supplement: Supplementary file 1 — se4c00488_si_001.pdf [file se4c00488_si_001.pdf]

Supporting Information

Rapid Detection of SARS-CoV-2 Variants Using ACE2-Based SERS Sensor Enhanced by CoVari Deep Learning Algorithms

Yanjun Yang [a]*, Jiaheng Cui [b], Dan Luo [c], Jackelyn Murray [d], Xianyan Chen [e], Sebastian Hülck [f], Ralph A. Tripp [d], Yiping Zhao [a]*

[a] *Department of Physics and Astronomy, The University of Georgia, Athens, GA, USA 30602*

[b] *School of Electrical and Computer Engineering, College of Engineering, The University of Georgia, Athens, GA, USA 30602*

[c] *Department of Statistics, The University of Georgia, Athens, GA, USA 30602*

[d] *Department of Infectious Diseases, College of Veterinary Medicine, The University of Georgia, Athens, GA, USA 30602*

[e] *Department of Epidemiology & Biostatistics, College of Public Health, The University of Georgia, Athens, GA, USA 30602*

[f] *Tec5USA Inc., Plainview, NY, USA 11803*

* Corresponding Author: E-mail: YanjunYang@uga.edu; zhaoy@uga.edu

Section S1. Characterization of the SERS substrate.

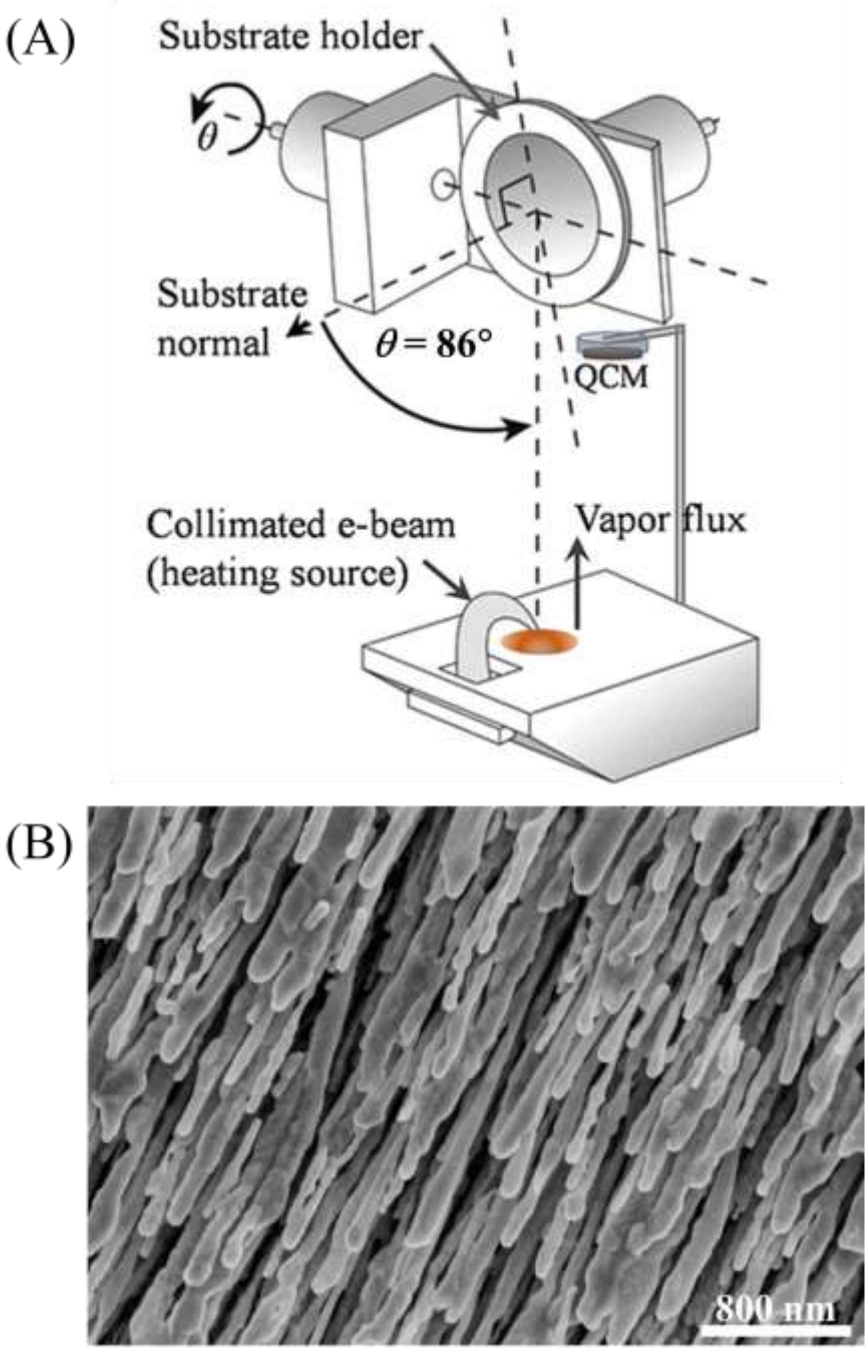

Figure S1. (A) Setup of the oblique angle deposition for silver nanorod (AgNR) arrays SERS substrate fabrication. (B) SEM image of AgNR@SiO_2 array SERS substrate.

Section S2. Additional information for the coronavirus detection.

Table S1. The number of SERS spectra collected from references and viruses of different concentrations.

Virus name	Total	Concentration (PFU/mL)										
		10^5	5×10^4	2.5×10^4	12,500	6,250	3,125	1,562	781	391	195	98
CoV-NL63	3259	233	258	254	291	270	276	273	355	351	347	351
SARS-CoV-2	3639	351	311	307	314	318	319	319	318	381	317	384
SARS-CoV-2 B1	3710	308	309	309	309	309	308	311	386	386	387	388
**Other spectra:**	SERS sensor: 734				Buffer: 729				CoV-229E: 474			

Table S2. SERS peak assignments for ACE2 immobilization.

Peak (cm^{-1})	Assignment	Peak (cm^{-1})	Assignment
558	S-S	1031	Phe
643	Tyr	1122	C-N
760	Trp	1235	Amide III
830	Tyr	1336	Trp, C_a-H (def)
854	Tyr	1447	C-H (def)
878	Trp	1552	Indole ring
939	N-C_α-C	1603	Tyr, Trp, Phe
1003	Phe	1670	Amide I

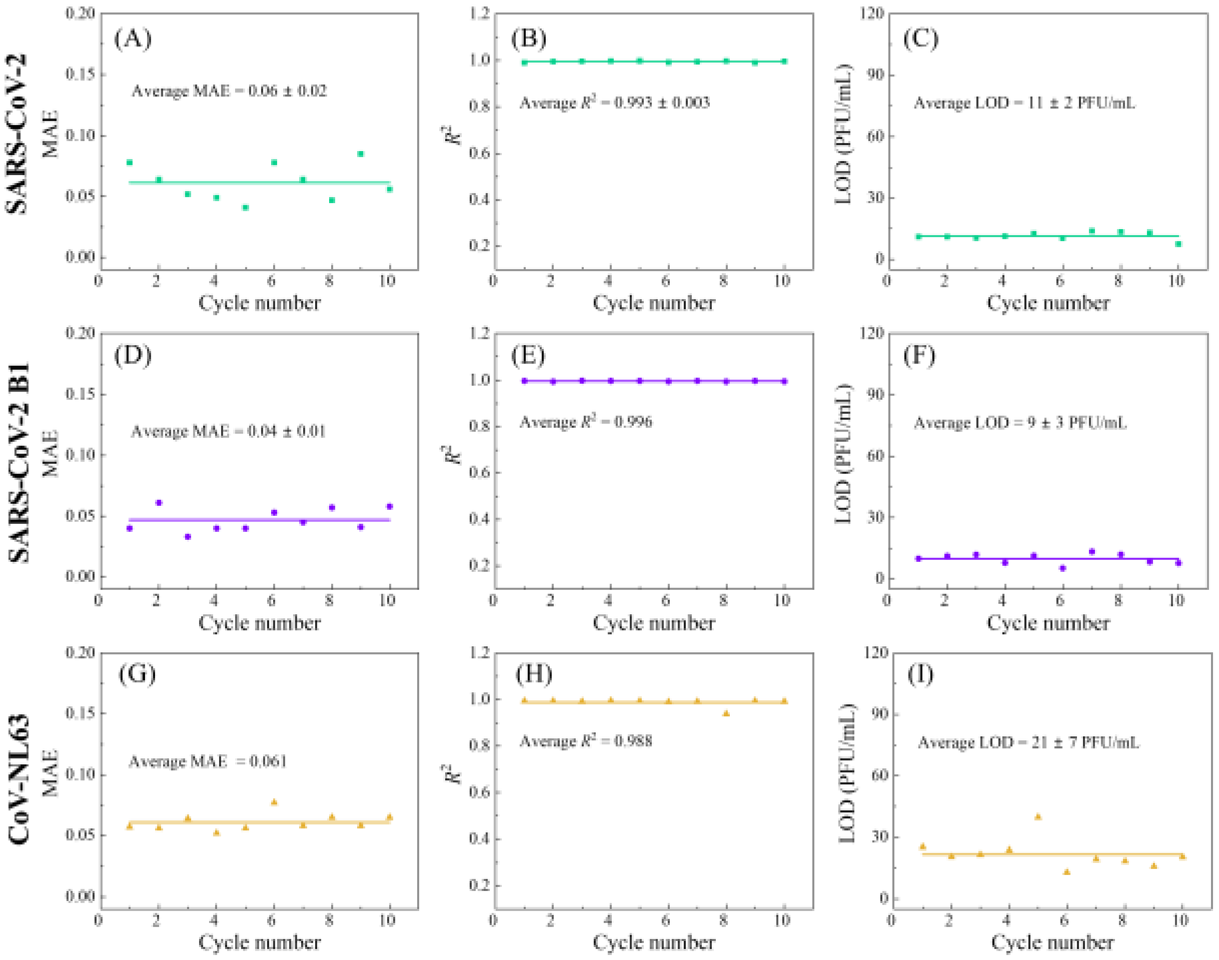

Figure S2. The detailed performance metrics of 10 cross-validations, including mean absolute errors (MAEs), R-squared (R^2), and limits of detection (LODs) for SARS-CoV-2 (A-C), SARS-CoV-2 B1 (D-F), and CoV-NL63 (G-I), respectively.

Section S3. Calculation of the permutation feature importance.

To comprehend the basis for the accurate classification and quantification results, it is useful to extract the discriminating SERS spectral features that the CoVari deep learning algorithm employs as decisive factors. Since the SERS peaks correspond to the vibration mode of different molecular compounds, the discriminating SERS spectral features might confer important biological information for many purposes. Given that the CoVari deep learning algorithm relies on spectral features for classifying and quantifying various coronaviruses, any changes in the important SERS peaks will dramatically affect the model performance. Therefore, permutation feature importance by shuffling the values of a specific feature and measuring the resulting decrease in the 'model's performance is used to assess the impact of each feature (i.e., wavenumber) in the spectrum,[1, 2] as illustrated in **Figure S3**. The process begins with calculating the original accuracy ($Accuracy_{ori}$) and original MAE (MAE_{ori}) based on the original test dataset. Then, for each wavenumber ($\Delta\nu$), a series of 100 "random permutation cycles" were performed. During every cycle, the corresponding SERS intensities for $\Delta\nu$ are randomly shuffled among all the specimens in the test dataset, while keeping the SERS intensities of other wavenumbers unchanged. The model is then evaluated again using the shuffled dataset, and the performance metric is calculated to obtain $Accuracy(\Delta\nu)$ and $MAE(\Delta\nu)$. The relative difference between the original metric value and the shuffled metric value indicates the importance of each feature. So, the feature importance of classification (FIC) is calculated by $FIC(\Delta\nu) = \frac{Accuracy_{ori} - Accuracy(\Delta\nu)}{Accuracy_{ori}}$, and the feature importance of regression (FIR) is calculated by $FIR(\Delta\nu) = -\frac{MAE_{ori} - MAE(\Delta\nu)}{MAE_{ori}}$. The averages of FIC and FIR, obtained from the 100 random permutation cycles for each wavenumber, were then plotted to visually depict their impact on the model's performance, as shown by the black and red curves in **Figure S2**. A larger decrease in performance suggests a higher importance of the spectral feature.

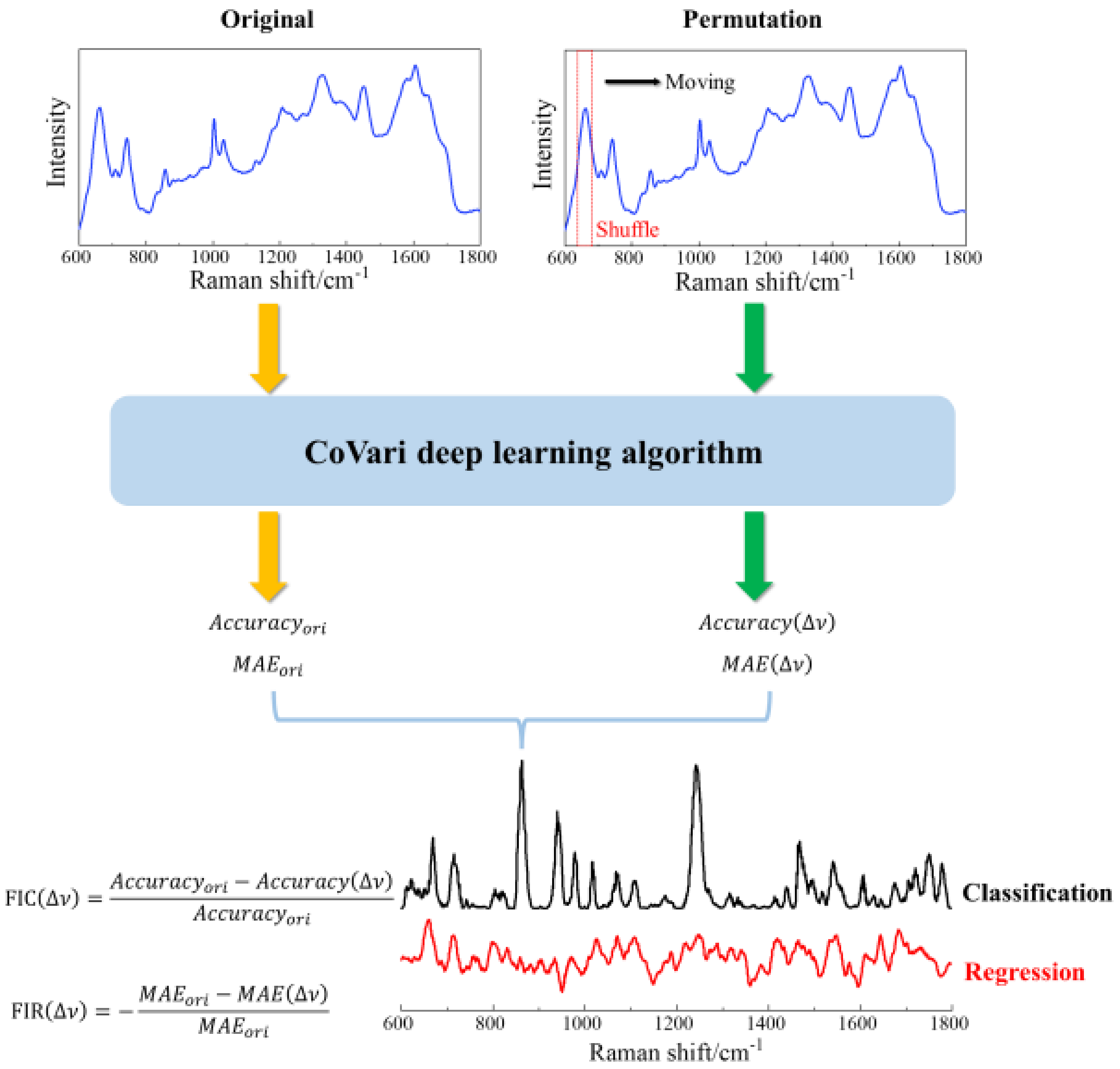

Figure S3. The flow chart illustrating the process of calculating feature importance of classification (FIC) and feature importance of regression (FIR).

Table S3. SERS peak assignments for feature importance of classification and regression.[3]

Classification		Regression	
**Peak**	**Peak assignment**	**Peak**	**Peak assignment**
667		659	Tyr
716		713	
863	Tyr	760	Trp
941	N-C_{α}-C	801	
980	Trp, Val	831	Tyr
1018		951	N-C_{α}-C
1072		1027	Phe
1110	C-N	1071	
1244	Amide III	1110	C-N
1465	C-H (def)	1150	
1542	Indole ring (Trp)	1187	
1608	Tyr, Trp, Phe	1220	
		1246	Amide III
		1288	
		1360	
		1399	
		1417	
		1465	C-H (def)
		1511	
		1547	Indole ring (Trp)
		1595	
		1644	
		1684	Amide I

Section S4. Calculation of the limit of detection (LOD).

Detailed values for $mean_{blank}$, SD_{blank} and LOB [4] are presented in **Tabel S4**.

Table S4. Values for the calculation of LOB

$mean_{blank}$ (PFU/mL)	SD_{blank} (PFU/mL)	LOB (PFU/mL)
0.005	0.0712	0.1222

Section S5. Traditional calibration curves for coronavirus detection.

Figure S4A plots the average SERS spectra with original peak intensity from the detection of SARS-CoV-2 (10^5 PFU/mL), SARS-CoV-2 B1 (10^5 PFU/mL), and CoV-NL63 (10^5 PFU/mL). The SERS peak intensities, I_{1608}, I_{1608}, and I_{1584} are plotted against virus concentrations $C_{\mathrm{SARS-CoV-2}}$, $C_{\mathrm{SARS-CoV-2\ B1}}$, and $C_{\mathrm{CoV-NL63}}$ in **Figures S4B-D** in a semi-log scale. All the calibration curves plotted by using interpolation of cubic B-spline show that SERS intensities increase monotonically with concentration, but the detailed relationships are not exactly the same. Actually, the SERS peak intensities do not show a distinguishable change at low virus concentrations. To estimate the LOD for each of these curves, according to the definition of the LOD from Eqs. (2) - (3) in the main text, the LODs for SARS-CoV-2, SARS-CoV-2 B1, and CoV-NL63 are estimated to be 991 PFU/mL, 1513 PFU/mL, and 13335 PFU/mL, respectively. These values are significantly higher than those determined by CoVari.

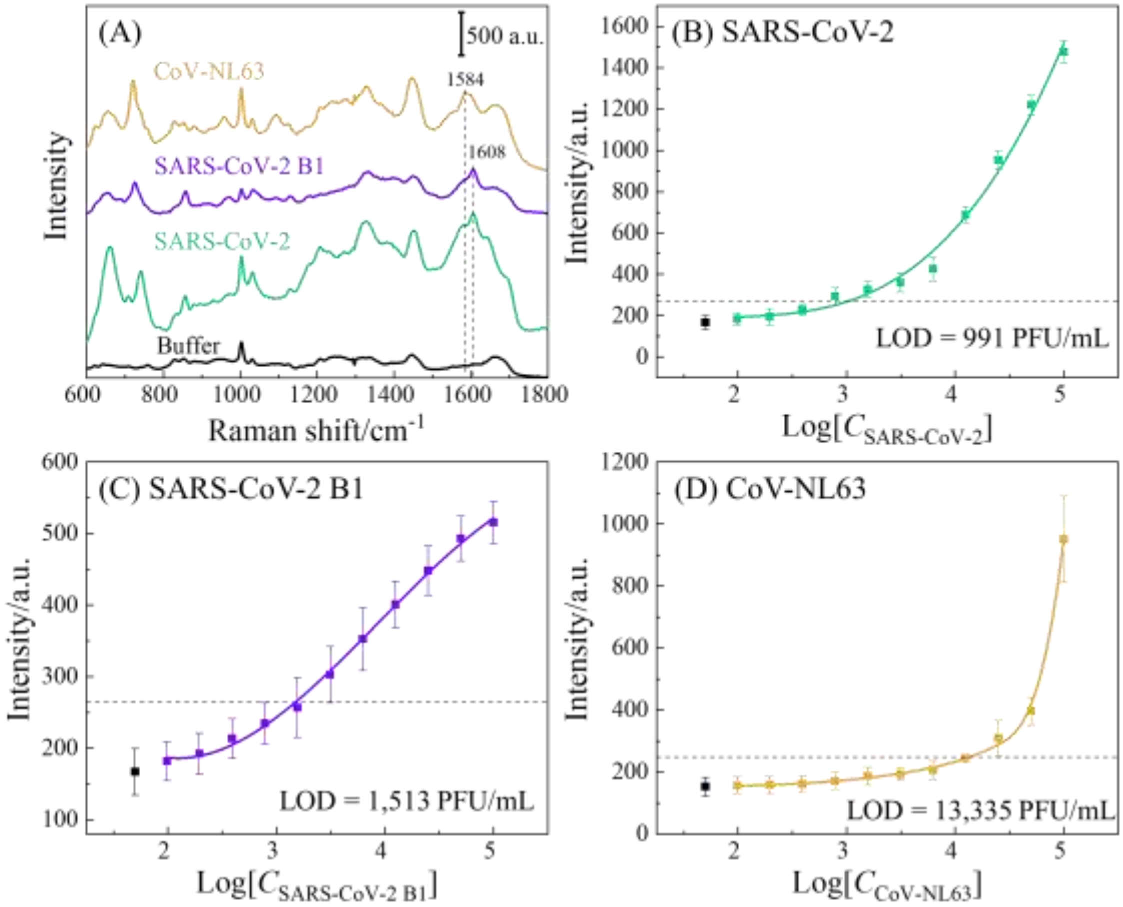

Figure S4. (A) The average SERS spectra of the detection of buffer, SARS-CoV-2 (10^5 PFU/mL), SARS-CoV-2 B1 (10^5 PFU/mL), and CoV-NL63 (10^5 PFU/mL). The SERS spectra have the original peak intensity without area normalization. (B) The semi-log plot of the SERS peak intensity I_{1608} versus SARS-CoV-2 concentration $C_{\text{SARS-CoV-2}}$. (C) The semi-log plot of the SERS peak intensity I_{1608} versus SARS-CoV-2 B1 concentration $C_{\text{SARS-CoV-2 B1}}$. (D) The semi-log plot of the SERS peak intensity I_{1584} versus CoV-NL63 concentration $C_{\text{CoV-NL63}}$. The black data points are from the SERS spectra of buffer, representing as background signal. The solid lines represent the best fitting.

Section S6. Additional results for unknown specimen test.

Table S5. Output accuracy of the CoVari for unknown viral concentration test.

Virus name	Concentration (PFU/mL)								
	**50,000**	**25,000**	**12,500**	**6,250**	**3,125**	**1,562**	**781**	**391**	**195**
CoV-NL63	100	100	98.7	98.7	99.1	99.7	96.5	100	75.0
SARS-CoV-2	100	100	100	100	100	99.4	100	99.2	92.0
SARS-CoV-2 B1	95.0	94.9	94.1	91.9	99.6	99.6	65.1	83.5	29.8

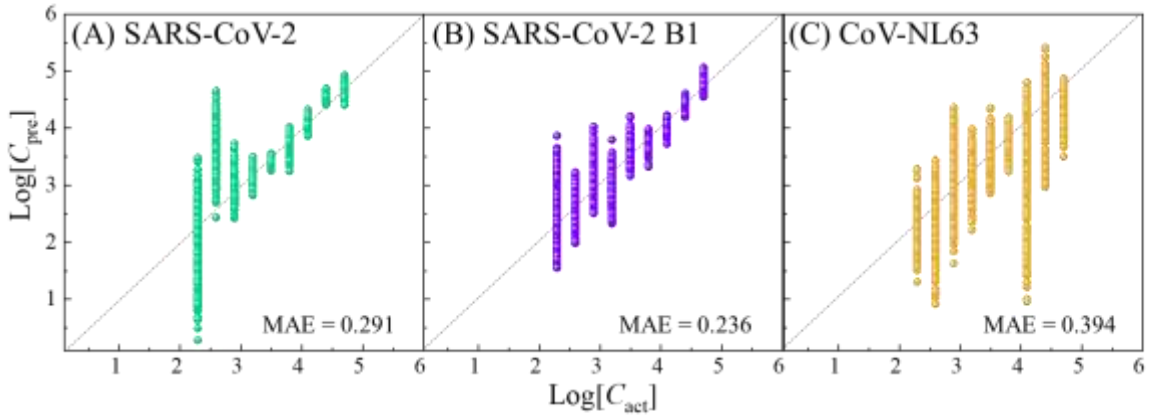

Figure S5. (A) Regression results of the CoVari for detection of three viruses with unknown concentrations in buffer for (B) SARS-CoV-2, (C) SARS-CoV-2 B1, (D) CoV-NL63. The *x*-axis is $\log_{10}(C_{act})$ of testing spectra, and *y*-axis is $\log_{10}(C_{pre})$. The dashed lines represent $\log_{10}(C_{act}) = \log_{10}(C_{pre})$. The unit of the concentrations is PFU/mL.

Section S7. Additional information on spike protein detection in saliva.

Table S6. The number of SERS spectra collected from references and spike proteins of different concentrations.

Name of spike protein		SARS-CoV-2 spike	SARS-CoV-2 spike (BA 2. 75. 2)	SARS-CoV-1 spike
Concentration	50 μg/mL	345	347	348
	10 μg/mL	346	343	343
	2 μg/mL	348	348	343
	0.4 μg/mL	346	342	347
	80 ng/mL	347	347	346
	16 ng/mL	346	344	347
	3.2 ng/mL	347	345	345
	640 pg/mL	347	342	348
	128 pg/mL	349	344	344
	25.6 pg/mL	344	342	344

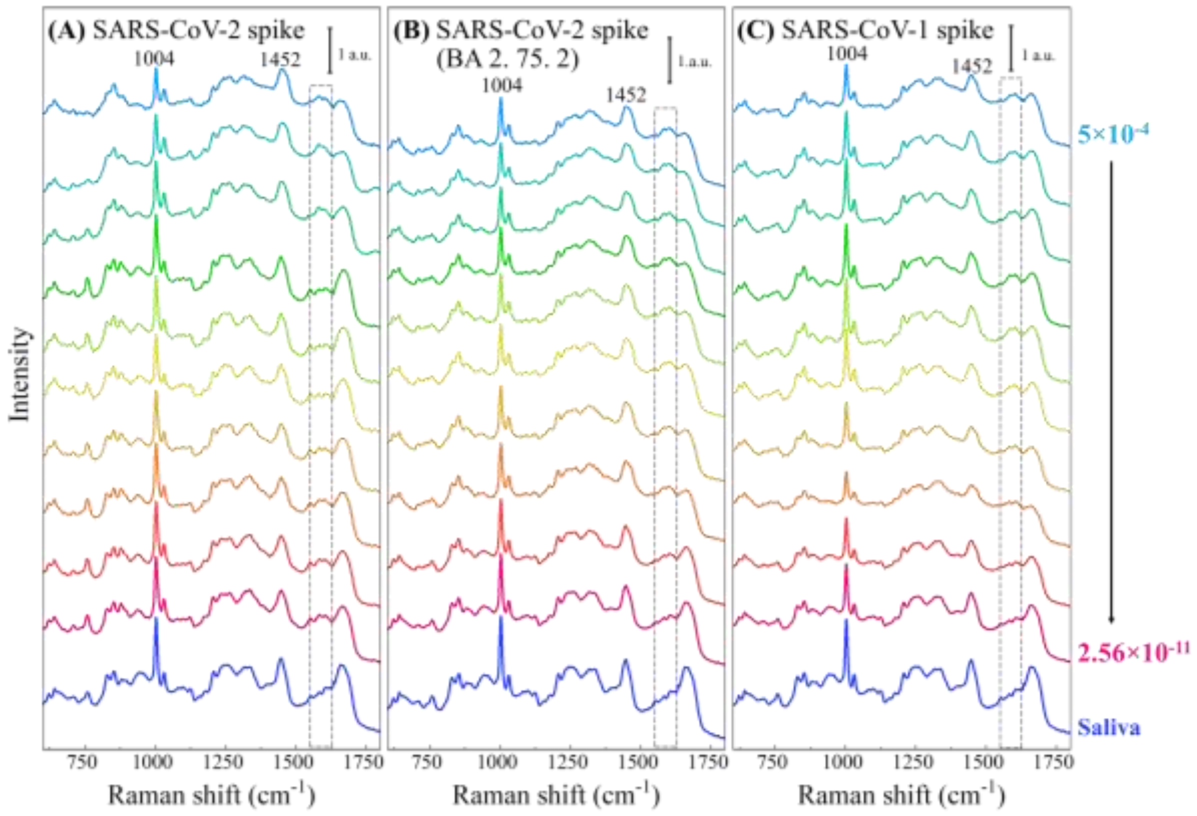

Figure S6. The average SERS spectra from (A) SARS-CoV-2 spike, (B) SARS-CoV-2 spike (BA 2. 75. 2), (C) SARS-CoV-1 spike at different concentrations in saliva. The concentrations are labeled beside the SERS spectra with the unit of g/mL.

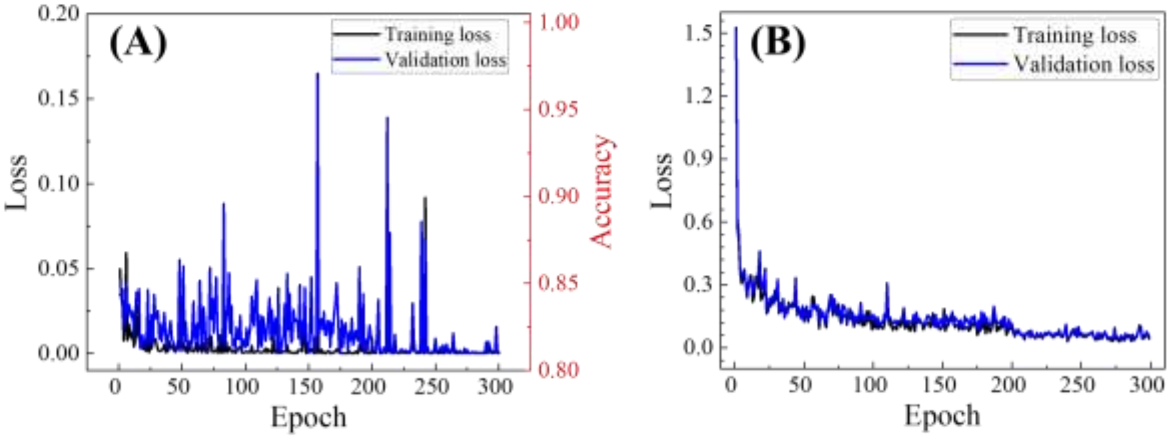

Figure S7. The plots of (A) the classification loss and accuracy and (B) the regression loss versus training epoch during the training and validation of the CoVari for spike proteins in saliva.

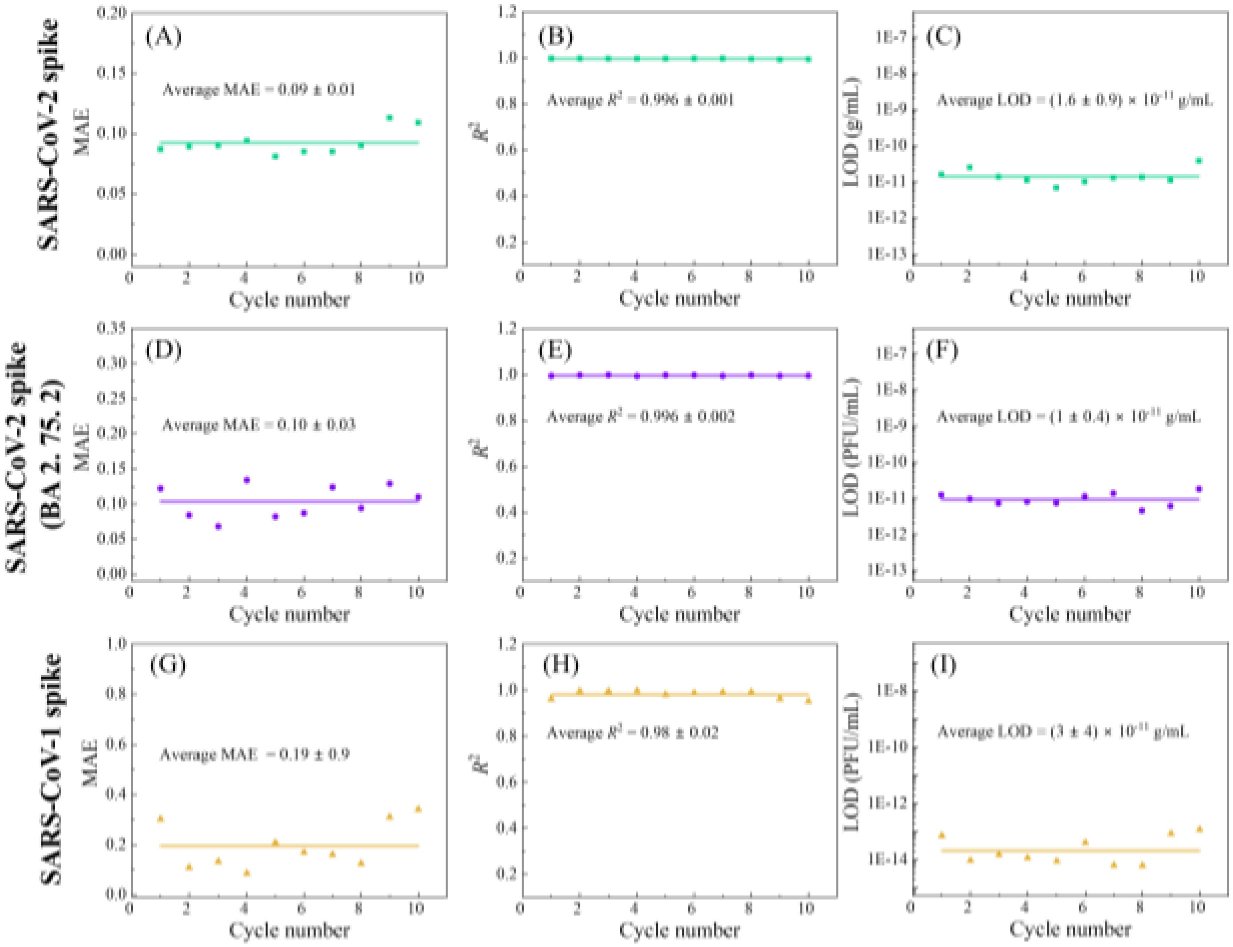

Figure S8. The detailed performance metrics of 10 cross-validations, including mean absolute errors (MAEs), R-squared (R^2), and limits of detection (LODs) for SARS-CoV-2 spike (A-C), SARS-CoV-2 spike (BA 2. 75. 2) (D-F), and SARS-CoV-1 spike (G-I), respectively.

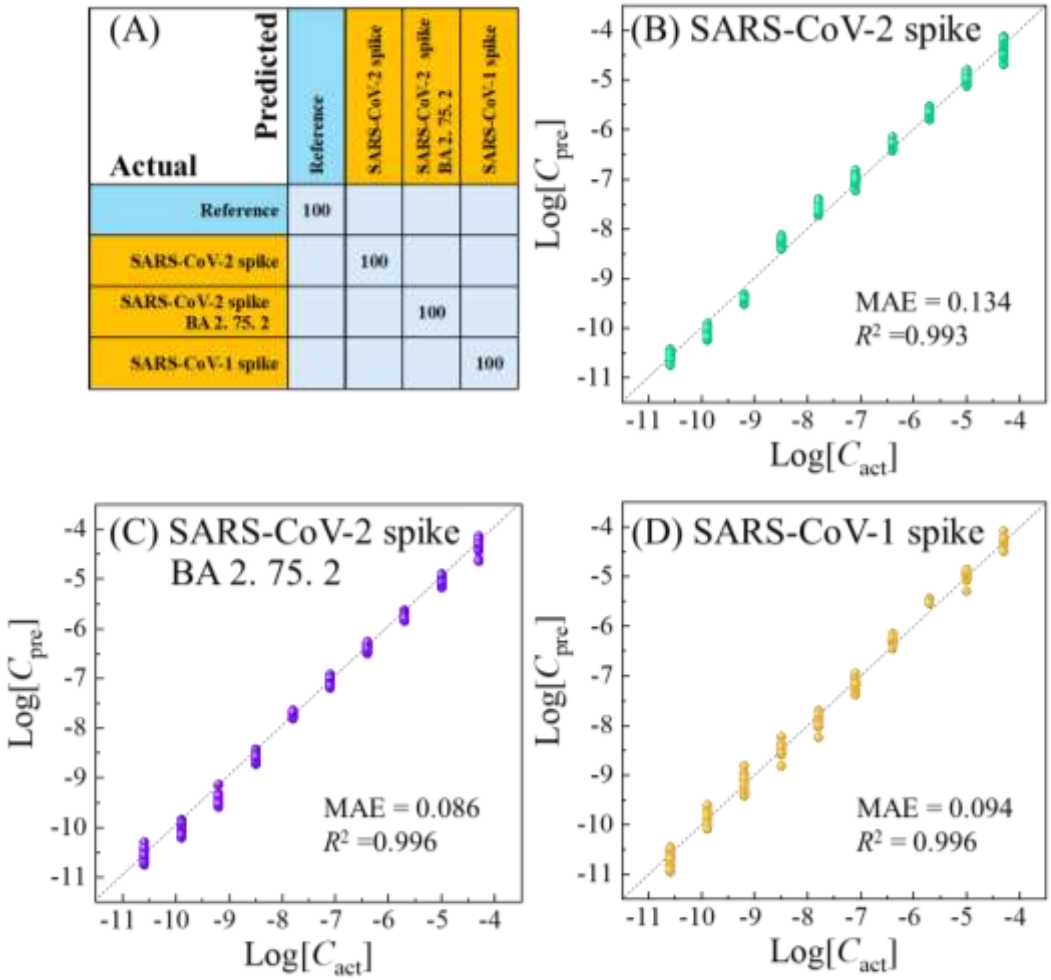

Figure S9. (A) The confusion matrix of the CoVari for detecting three coronavirus spike proteins and references. Regression results of the CoVari for (B) SARS-CoV-2 spike, (C) SARS-CoV-2 spike (BA 2. 75. 2), (D) SARS-CoV-1 spike. The *x*-axis is $\log_{10}(C_{act})$ of testing spectra, and *y*-axis is $\log_{10}(C_{pre})$. The dashed lines represent $\log_{10}(C_{act}) = \log_{10}(C_{pre})$. The unit of the concentrations is g/mL.

References:

1. Altmann, A.; Toloşi, L.; Sander, O.; Lengauer, T., Permutation importance: a corrected feature importance measure. *Bioinformatics* **2010,** *26* (10), 1340-1347.
2. Breiman, L., Random Forests. *Machine Learning* **2001,** *45* (1), 5-32.
3. Rygula, A.; Majzner, K.; Marzec, K. M.; Kaczor, A.; Pilarczyk, M.; Baranska, M., Raman spectroscopy of proteins: a review. *Journal of Raman Spectroscopy* **2013,** *44* (8), 1061-1076.
4. Armbruster, D. A.; Pry, T., Limit of blank, limit of detection and limit of quantitation. *Clinical Biochemist Reviews* **2008,** *29* (Suppl 1), S49-S52.
